# Molecular and Immunological Properties of a Chimeric Glycosyl Hydrolase 18 Based on Immunoinformatics Approaches: A Design of a New Anti-*Leishmania* Vaccine

**DOI:** 10.1021/acsptsci.4c00341

**Published:** 2024-12-31

**Authors:** José Ednésio da Cruz Freire, André Nogueira Cardeal dos Santos, Andrelina Noronha Coelho de Souza, Ariclécio Cunha de Oliveira, Roberto Nicolete, Bruno Lopes de Sousa, João Hermínio Martins da Silva, Yuri de Abreu Gomes Vasconcelos, Isaac Neto Goes da Silva, Paula Matias Soares, Maria Izabel Florindo Guedes, Vânia Marilande Ceccatto

**Affiliations:** † Superior Institute of Biomedical Sciences, State University of Ceará, Fortaleza, Ceará 60714-903, Brazil; ‡ Oswaldo Cruz Foundation (Fiocruz Ceará), Eusébio, Ceará 61773-270, Brazil; § Department of Veterinary Sciences, State University of Ceará, Fortaleza, Ceará 60714-903, Brazil; ∥ Biotechnology and Molecular Biology Laboratory, State University of Ceará, Fortaleza, Ceará 60714-903, Brazil

**Keywords:** vaccines, chitinases, epitope mapping, in silico analysis, anti-*Leishmania*

## Abstract

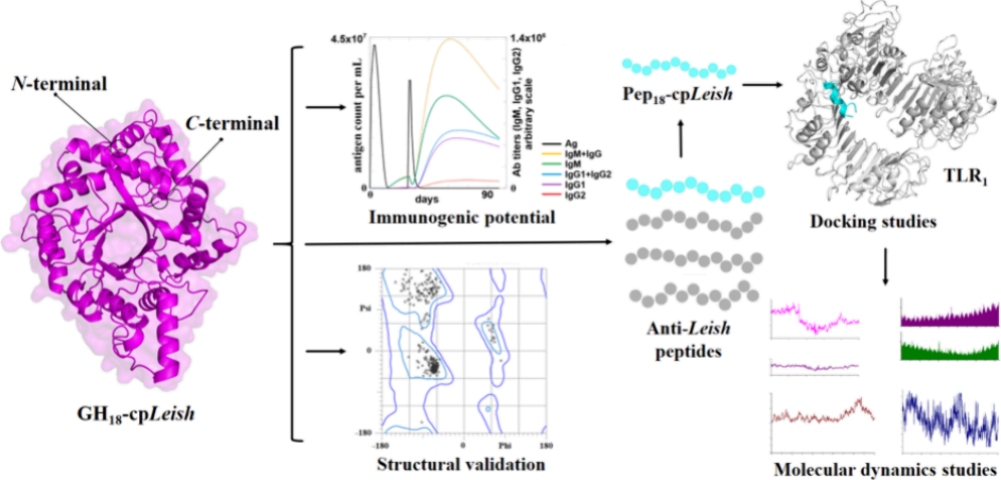

Leishmaniasis is a chronic inflammatory zoonotic illness caused by protozoan flagellates belonging to the *Leishmania* genus. Current data suggest that over 1 billion people worldwide are susceptible to infection, primarily in tropical and subtropical countries, where up to 2 million new cases are reported annually. Therefore, the development of a vaccine is crucial to combating this disease. This study employed immunoinformatics approaches to design a multiepitope anti-*Leishmania* vaccine, GH_18_-cp*Leish*, based on a cluster of six glycosyl hydrolases 18. We identified six helper T lymphocyte (HTL) epitopes and twenty-six cytotoxic T lymphocyte (CTL) epitopes with IC_50_ values <50 nM, indicating high affinity. Additionally, we also identified 20 continuous and twenty-six discontinuous B-cell epitopes. Analysis for allergenicity and toxicity showed no potential to induce these phenomena. All data obtained from in silico tools suggest that physicochemical and biological studies indicate that the GH_18_-cp*Leish* chimeric protein is a promising candidate for an anti-*Leishmania* vaccine. Docking analysis showed that the Pep_1_-cp*Leish*::TLR_1_, Pep_1_-cp*Leish*::TLR_2_, Pep_1_-cp*Leish*::/TLR_3_, and Pep_1_-cp*Leish*::/TLR_4_ complexes maintained a stable form. The best interaction cluster score was observed in the complex Pep_1_-cp*Leish*::TLR_2_ (center = −622.6 and lowest energy = −841.7 kcal.mol^–1^) followed by the complexes Pep_1_-cp*Leish*::TLR_4_ (center = −590.3 and lowest energy = −590.3 kcal.mol^–1^), Pep_1_-cp*Leish*::TLR_3_ (center = −589.1 and lowest energy = −657.0 kcal.mol^–1^), and Pep_1_-cp*Leish*::TLR_1_ (center = −504.1 and lowest energy = −602.9 kcal.mol^–1^), respectively. This study suggests that GH_18_-cp*Leish* may be suitable for constructing second-generation anti-*Leishmania* and even third-generation vaccines, given that its gene sequence is optimized for this purpose.

Leishmaniasis is a zoonotic chronic inflammatory illness that affects humans and is caused by members of the genus *Leishmania*. They are transmitted through the bites of parasite-infected sandflies, mainly those belonging to the genera *Phlebotomus* and *Lutzomyia*. For decades, the number of reported leishmaniasis cases has posed a severe public health problem, particularly in countries such as India, Bangladesh, Sudan, South Sudan, Ethiopia, and Brazil.^1,2^ These countries collectively account for over 90% of all reported cases worldwide. At present, the available therapeutic options for leishmaniasis remain limited due to the prevalence of numerous side effects. Furthermore, specific treatments, such as antibiotics, contribute to the selection of increasingly resistant parasites.^3−5^ Furthermore, as of the present date, there is no record of any anti-*Leishmania* vaccines being available for clinical or commercial use in humans.^6−10^ Confronted with this severe public health crisis, significant efforts have been undertaken to develop an effective anti-*Leishmania* vaccine that can be made accessible to the population. The advancement of effective vaccines for leishmaniasis has faced numerous challenges, including inadequate funding, the absence of commercial incentives, insufficient overall support for public health, unreliable predictive models, and researchers’ hesitance to pursue uncertain paths.^11−15^

Prior studies have identified a substantial cluster of significant immunoreactive orthologous proteins referred to as chitinases or glycosyl hydrolases of family 18 (GH_18_), originating from various organisms, including species within the *Leishmania* genus.^16−20^ These proteins contain epitopes that antibodies can recognize in mammals, including humans. Throughout the infection process, the cellular immunity triggered by the Th1/Tc1 (type 1 helper and cytotoxic T-cells) response is considered a significant mediator of resistance against *Leishmania* species.^21^ Furthermore, individuals affected by leishmaniasis also demonstrated elevated levels of proinflammatory cytokines, including interferon-gamma (IFNγ) and tumor necrosis factor-alpha (TNFα),^22,23^ heightened Th2 cell activity,^24^ persistent inflammatory lesions,^21,24^ and increased interleukin-4 receptor alpha (IL-4Rα) signaling in dendritic cells.^25^ Additionally, indexes of interleukin (IL)-1β, IL-4, IL-5, IL-10, IL-13, IL-17, and IL-23 also appear to be elevated in individuals affected by cutaneous and mucocutaneous leishmaniasis.^22,26−29^ On the flip side, Toll-like receptors (TLRs) present in resident cells (e.g., macrophages, keratinocytes, mast cells, Langerhans cells, and others) can recognize pathogen-associated molecular patterns and trigger the phagocytosis of pathogens and opsonized particles.^21^ Collectively, these cells can prompt the expression of diverse cytokine receptors and collaborate with tissue cells to generate chemokines, initiating immune responses linked to innate and acquired immune systems.

In recent years, immunoinformatics approaches have been developed to predict antigenicity based on peptides/proteins,^30−33^ with an accuracy rate of up to 82% in predicting known protective antigens.^34^ As a result, numerous vaccines have been designed using this in silico tool, including antiviral vaccines,^7,35^ antibacterial,^36,37^ antimalaria,^38^ antitoxoplasmosis,^39^ and anticryptosporidiosis,^40^ among others.

This study employed bioinformatics and immunoinformatics approaches to predict a chimeric protein’s physicochemical and immunogenic properties (GH_18_-cp*Leish*) crafted from GH_18_ proteins of *Leishmania* spp. Our objective was to perform a thorough in silico analysis of the structural features and immunogenic potential of the peptides derived from GH_18_-cp*Leish*, capable of eliciting immune responses mediated through TLR_1_, TLR_2_, TLR_3_, and TLR_4_. These analyses are intended to develop a novel anti-*Leishmania* vaccine (GH_18_-cp*Leish*) based on peptides that target human leishmaniasis and other animals.

## Methods

### Retrieval of Bait Sequences and GH_18_-cp*Leish* Gene Design

The amino acid sequences of chitinases expressed in*Leishmania braziliensis* (access: lbz: LBRM_16_0800), *L. donovani* (access: ldo: LDBPK_160790), *L. infantum* (access: lif: LINJ_16_0790), *L. major* (access: lma: LMJF_16_0790), *L. mexicana* (access: lmi: LMXM_16_0790) and *L. panamensis* (access: lpan: LPMP_160760) were downloaded in.*fasta* format from Kyoto Encyclopedia of Genes and Genomes (https://www.genome.jp/kegg/). For this purpose, the sequence encoding GH_18_ is from *L. braziliensis* (accession: lbz: LBRM_16_0800) was used as a reference and bait to identify all correlated GH_18_ isoforms in other *Leishmania* species. Subsequently, we utilized the algorithm provided by the CLC Sequence Viewer 8.0 software package (www.clcbio.com) to perform multiple alignments and predict a consensus sequence (GH_18_-cp*Leish*). The consensus sequence (theoretical sequence) represented by nucleotides or amino acids, as shown in Figure S1a,b, was designed based on amino acid being the one that occurs most frequently at that position in the different native sequences. The proposed multiepitope anti-*Leishmania* vaccine features ligand-linked epitopes (AAY, KK, and GGGS) and the adjuvant P9WHE3 (50S ribosomal protein L7/L12 – *Locus* RL7_MYCTU) in the *N*-terminal region of the multiepitope protein. Immediately following the adjuvant, in the upstream direction, the pan HLA DR-binding epitope (PADRE: AKFVAAWTLKAAA) and the GGGS linker were added. Subsequently, CTLs and HTLs were introduced, “data not shown”. Finally, in the *C*-terminal region, a His tag tail (hexahistidine) was fused.

The gene encoding GH_18_-cp*Leish* was designed and then codons-optimized and refined according to the recommendations of the online gene optimization database – Jcat Server (http://www.jcat.de/) and the method described by Bai et al.^41^ For this purpose, the amino acid sequence of GH_18_-cp*Leish* was initially used to obtain an appropriate gene sequence that would be more easily recognized, processed, and expressed in *Komagataella pastoris* strains (homotypic synonym *Pichia pastoris*), given that the gene sequences used in this work are native to *Leishmania* species. The codon-optimized gene sequence was then subjected to a manual refinement process, as described by Bai and collaborators. This additional step is necessary because, in many cases, the gene obtained after codon optimization is not yet fully suitable for specific species such as *K. pastoris*, requiring adjustments to some of the remaining codons. The RNA secondary structures (open reading frame) were predicted using the Vienna RNA Secondary Structure Prediction Server (http://rna.tbi.univie.ac.at/).

To identify signal peptides (SP) and potential cleavage sites, all sequences were submitted for prediction on the SignalP 6.0 server (https://services.healthtech.dtu.dk/services/SignalP-6.0/) and the XtalPred-RF server (https://xtalpred.godziklab.org/XtalPred-cgi/xtal.pl), respectively. The mature GH_18_-cp*Leish* sequence obtained after signal peptide (SP) evaluation was used in all subsequent analyses by previous investigations.^42−45^

### Physicochemical Characterization and Analysis of Conserved Domains

Using the XtalPred-RF Server, the physicochemical properties of GH_18_-cp*Leish*, including the theoretical molecular weight (Mr), isoelectric point (pI), aliphatic index (AI), and hydrophobicity index (GRAVY), were assessed. The estimated half-life in mammalian reticulocytes, yeast, and *Escherichia coli* was predicted using the Expasy ProtParam Server (https://web.expasy.org/protparam/). The prediction of disordered regions was conducted using the ADOPT – Attention Disorder Predictor Server (https://adopt.peptone.io/). The analysis of the presence or absence of disulfide bridges was carried out using the DiANNA 1.1 Web Server (https://bioinformatics.bc.edu/clotelab/DiANNA/). Phosphorylation sites were predicted by PhosTryp–Phosphosite Predictor in *Trypanosomatidae* (http://phostryp.bio.uniroma2.it/). Predictions of glycosylation sites were performed using the Net*N*Glyc 1.0 (https://services.healthtech.dtu.dk/services/NetNGlyc-1.0/) and Yin*O*Yang 1.2 servers (https://services.healthtech.dtu.dk/services/YinOYang-1.2/)^46^ to identify type *N*-linked and *O*-linked glycosylations, respectively. Domain analysis and linker predictions were carried out using the Conserved Domains Database Server (https://www.ncbi.nlm.nih.gov/Structure/cdd/cdd.shtml).

### 3D Structure Prediction and Analysis

To identify suitable templates for predicting the three-dimensional structure of GH_18_-cp*Leish*, we followed the approach described by Monteiro-Júnior and collaborators,^47^ using the RCSB - Protein Data Bank Server (https://www.rcsb.org/) as the search database for structural templates. A theoretical 3D model of GH_18_-cp*Leish* was predicted using the Robetta Server software (https://robetta.bakerlab.org/). Then, the model was improved by adjusting the arrangement of Φ–ψ dihedral angle pairs and side chains and finally further refined through energy minimization using the Coot 0.9.6 package^48^ and the Yasara - Yet Another Scientific Artificial Reality Application package,^49^ respectively. The quality of the refined model was evaluated using Molprobity (http://molprobity.biochem.duke.edu/), QMEAN (https://swissmodel.expasy.org/qmean/), VERIFY 3D (https://www.doe-mbi.ucla.edu/verify3d/), and ProSA-Web (https://prosa.services.came.sbg.ac.at/prosa.php) Servers.

### Prediction of Immunogenic, Allergic, and Toxic Properties

To assess the potential for immunogenicity of GH_18_-cp*Leish*, we initially searched the available human proteins data set using the BLASTp Server (https://blast.ncbi.nlm. nih.gov/Blast.cgi) to ensure that this protein exhibits no similarity to any human protein. Then, the potential immunogenicity of active GH_18_-cp*Leish* (with no signal peptide) was predicted by the VaxiJen 2.0 Server (https://www.ddg-pharmfac.net/vaxijen/VaxiJen/VaxiJen.html) using a threshold cutoff value of −0.5. Analyses of linear (continuous) and conformational (discontinuous) B-cell epitopes were conducted using the ABCpred Server (http://crdd.osdd.net/raghava/abcpred/) and the ElliPro Server (http://tools.iedb.org/ellipro/), respectively.

The prediction of epitopes processed by major histocompatibility complex class I (MHC-I) and class II (MHC-II) was performed using data from the IEDB database (https://www.iedb.org/). The search for peptidic epitopes capable of binding to MHC-I was parametrized by selecting nine or 10-*mers*, utilizing a set of 54 alleles integrated into an artificial neural network (ANN). In contrast, the search for MHC-II binding epitopes involved selecting the complete human HLA reference set (27 alleles) and choosing 16-*mers*. Both types of searches aimed to identify the IC_50_ values.

The in silico profile of the cell-mediated and humoral immune response in mammals following vaccine administration based on the GH_18_-cp*Leish* was assessed using the C-ImmSim Server (https://wwwold.iac.rm.cnr.it/~filippo/c-immsim/). The simulation was parametrized as follows: (I) Vaccines were administered without LPS, (II) Two administrations were given at an interval of 30 days, (III) Set to 1, 84, and 168 - with a time step of 1 indicating the injection at time 1/4 0, and (IV) simulation steps were adjusted to 1050. Default parameters were maintained for all other random seeds, host alleles, and simulation volumes.

The allergenicity and toxicity potential of GH_18_-cp*Leish* were predicted using the AlgPred 2.0 Server (https://webs.iiitd.edu.in/raghava/algpred2/), AllergenFP 1.0 Server (https://www.ddg-pharmfac.net/AllergenFP/), and ToxinPred Server (http://crdd.osdd.net/raghava/toxinpred/). AlgPred and AllergenFP predict potentially allergenic peptides, while the ToxinPred server predicts potentially toxic epitopes. Analysis performed by AlgPred employed a hybrid approach (immunoglobulin E epitope + SVMc + MAST + ARPs BLAST) with a chosen threshold of −0.4. AllergenFP used an approach based on the physicochemical properties of the peptides for its analysis. Similarly, toxicity analysis by ToxinPred also used an approach based on the physicochemical properties of the peptides.

### Docking and Molecular Dynamics Studies

The peptide with the highest immunogenic potential, obtained from the final refined model of GH_18_-cp*Leish*, was subjected to molecular docking predictions with human Toll-like receptors (TLR_1_, TLR_2_, TLR_3_, and TLR_4_). The receptor’s structure was obtained from RCSB Protein Data Bank with the following IDs: 6NIH, 6NIG, 7WFI, and 3FXI, respectively.^50,51^ The interactions between the anti-*Leish* peptide (Pep_1_-cp*Leish*: ^402^RGSGGNVNSDNAYDCP^418^) and TLRs were simulated using the ClusPro protein–protein docking Server (https://cluspro.org/help.php). For the docking studies, the following parameters were used: (I) Simulations of PIPER-based rigid body docking with an exhaustive sampling of the conformational space on a dense grid, (II) Clustering of the 1000 lowest energy docked structures using interface root-mean-square deviation (iRMSD) as the distance measure, and (III) Refinement of complex molecules located at cluster centers through minimization of their energy. In addition, the binding free energy (Δ*G* in kcal/mol^–1^) of the docked complexes was also obtained from the PRODIGY Webserver (https://rascar.science.uu.nl/prodigy/). It was employed in this study, and we used the equation Δ*G* = *RT*ln(*K*_d_) to calculate the dissociation constant *K*_d_, where *R* is the ideal gas constant (kcal K^–1^.mol^–1^), and *T* is the temperature (K).

In order to represent the potential of Pep_1_-cp*Leish* in interactions with TLRs, a complex was randomly selected and submitted to stability tests via molecular dynamics (MD) analysis using the GROMACS package, version 2019.5.^52^ The electroneutrality of the complex was ensured by adding Na^+^ ions at the appropriate concentration.^45,53^ System solvation was maintained using SPC/E (extended simple point charge model) water molecules in a periodic box (90 × 90 × 90 nm^3^ volume), containing a buffer to allow substantial conformational fluctuations during MD simulations, as described in previous publications. MD simulations were parametrized using the CHARMM36 force field, with energy minimization (2000 steps of steepest descent followed by a 200 ps MD simulation) performed prior to the start of the MD simulations to eliminate initial steric clashes.^53^ MD simulations were conducted under constant pressure, established by anisotropic diagonal position scaling with a time step of 0.002 ps.^45,53^ Electrostatic interactions were evaluated using the PME (Particle Mesh Ewald) method, with short-range cutoffs of 1.2 nm.^54^ The system temperature was gradually increased from 100 to 310 K at 1 bar pressure over 1000 ps. The Berendsen weak-coupling algorithm^55^ was employed with a coupling time constant of 0.2 ps. The LINCS algorithm^56^ was used to constrain the equilibrium distances between all bonds, allowing only internal motions of bending and torsion during the MD simulations. Finally, 100 ns MD simulations were performed in triplicate under the same conditions as the equilibration procedure.

Two-dimensional representations of the receptor-protein complexes based on the theoretical binding energy value were generated using the PIC: Protein Interactions Calculator Server (http://pic.mbu.iisc.ernet.in/job.html) and Arpeggio Server (https://biosig.lab.uq.edu.au/arpeggioweb/). The three-dimensional structures were built using the software PyMol Molecular Graphics System, version 1.7.4 (Schrodinger, LLC).

## Results

### Analysis of mRNA Sequence and GH_18_-cp*Leish* Design

Predictions obtained from the gene encoding GH_18_-cp*Leish* showed that the codon adaptation index (CAI) was 1.0 (100%), indicating optimal conditions for expression in *K. pastoris* (Figure S1b). The analysis of the GC content revealed a composition of 45.89% and AT content comprising 54.11%. In addition, the gene’s open reading frames exhibit a secondary structure with a minimum free energy (MFE) Δ*G* of −413.50 kcal.mol^–1^ (Figure S2a). The free energy of the thermodynamic ensemble equals −439.56 kcal.mol^–1^, and the centroid secondary structure displays a minimum free energy of −349.88 kcal.mol^–1^ (Figure S2b). Figure S2c displays a mountain plot representing the minimum free energy (MFE) structure. Additionally, Figure S2d showcases the thermodynamic ensemble of RNA structures, the centroid structure, and the positional entropy for each position.

Primary sequence analysis of native GH_18_ chitinase (*Leishmania braziliensis*, *L. donovani*, *L. infantum*, *L. major*, and *L. mexicana*) using SignalP 6.0 and XTalPred-RT servers showed that these full-length proteins are composed of 457 amino acids, except *L. panamensis*, which consists of 458 amino acids. All of these sequences contain an *N*-terminal signal peptide made up of 23 amino acids, which, after being cleaved off, results in mature polypeptides formed by 434 amino acid residues, except for *L. panamensis*, which has 435 amino acid residues (Table 1). Similar to native sequences from *Leishmania* spp., the consensus sequence of chitinase (GH_18_-cp*Leish*, Figure S1a), after prediction, displays a 457 amino acid full-length polypeptide that contains a 23 amino acid long *N*-terminal signal peptide, which is cleaved off to form the mature protein with 434 amino acid residues. The data suggest a cleavage site between Ser^23^ and Ala^24^ residues with a mean *S* score of 0.835 and a discrimination score of 0.668 (Table 1). A more detailed analysis of the *N*-terminal region of the GH_18_-cp*Leish* protein (^1^**MVQRSALVQLACLVAVLHSSCLS**APLS···L^457^, Figure S1a) reveals different characteristics typical of a signal peptide, including (I) *Positively Charged Region*: Typically, the first positions of the signal peptide contain positively charged amino acids, such as arginine (R) and lysine (K), which interact with the membrane. In this particular case, the sequence contains residue R^4^; (II) *Hydrophobic Region*: It has a central hydrophobic region (^7^LVQLACLVAVL^17^) that interacts with the lipid bilayer of the endoplasmic reticulum membrane, facilitating the insertion of the protein; (III) *Cleavage Site*: A specific cleavage sequence near the end of the signal peptide is where the protease cleaves to release the mature protein. In this particular case, the signal peptide ends before ^24^APLS···L^457^ (as indicated in bold), where cleavage occurs.

**Table 1:** Predicted Signal Peptide Cleavage Sites for All Chitinases from *Leishmania* spp., Available at KEGG: Kyoto Encyclopedia of Genes and Genomes (https://www.genome.jp/kegg/)^a^

species	access	*S*-score	*D*-score	cutoff	signal peptide cleavage point	amino acids (full-length)	amino acids (mature protein)
*Leishmania braziliensis*	lbz: LBRM_16_0800	0.924	0.748	0.450	LSCLG - APLST	457	434
*Leishmania donovani*	ldo: LDBPK_160790	0.919	0.697		SSCLS - ALLSS	457	434
*Leishmania infantum*	lif: LINJ_16_0790	0.919	0.697		SSCLS - ALLSS	457	434
*Leishmania major*	lma: LMJF_16_0790	0.881	0.761		SSCLS - APLSS	457	434
*Leishmania mexicana*	lmi:LMXM_16_0790	0.781	0.651		SSCVS - TPVSS	457	434
*Leishmania panamensis*	lpan: LPMP_160760	0.953	0.798		LSCLG - APLST	458	435
**GH**_**18**_**-cp*****Leish***	**Chimera protein**	**0.835**	**0.668**		**SSCLS - APLSS**	**457**	**434**

a The *N*-terminal signal peptide cleavage site of chitinases was predicted using the SignalP and XtalPred-RT servers. Most subtypes are cleaved close to a highly conserved alanine (A). Only one subtype (*L. mexicana*) shows a substitution of alanine for a threonine (A → T).

Additionally, obtaining and evaluating this information in a target sequence are often predicted using different approaches based on the software or server used for this function. In general, these approaches use mathematical models based on artificial neural networks (ANNs) and hidden markov models (HMMs); however, it is also possible to find mathematical and statistical models capable of making these predictions.

### Analysis of Physicochemical Characteristics and Conserved Domains

In silico analysis revealed that the GH_18_-cp*Leish* protein has a molecular mass of 50.34 kDa, a mature protein (without the signal peptide) of 47.93 kDa, and an isoelectric point (pI) of 6.06, making it an acidic protein. Predictions of the aliphatic index indicate that this protein could be considered stable based on a value of 81.01 obtained in a broad spectrum of temperatures. The GRAVY value of mature GH_18_-cp*Leish* was predicted to be −0.29, suggesting a high probability that this protein interacts with water molecules.

Using the ProtParam Server, we estimated that the half-life from GH_18_-cp*Leish* is 4.4 h (mammalian reticulocytes, *in vitro*), >20 h (yeast, *in vivo*), and >10 h (*Escherichia coli*, *in vivo*). The search for possible disulfide bonds and phosphorylation sites was predicted by the DiANNA 1.1 Web and PhosTryp Servers and suggested the existence of two CyS-SCy (cystines) interactions formed by the residues (Cys^108^-Cys^416^) and (Cys^109^-Cys^346^) and the presence of 16 potential phosphorylation sites being 13 serine and three threonines (Figure S1b and Table S1), respectively.

Using the Net*N*Glyc 1.0 and Yin*O*Yang 1.2 servers, the hotspots exposed to glycosylation machinery were identified, and therefore, GH_18_-cp*Leish* was a glycosylated protein. The predictions suggest that 12.9% of the amino acids are *O*-glycosylated (Ser, Thr, or Tyr), while only 0.46% are *N*-glycosylated (Asn), as indicated in Figure S1b. Using the CDD Server, we demonstrate that GH_18_-cp*Leish* belongs to the glycosyl hydrolase family 18 (GH_18_), based on an *E*-value of 7.81^–40^, Bit-Score of 144.50, and interval (amino acids) of 38 – 373 observed.

### Model Construction, Refinement, and Stereochemical Evaluation

**Figure 1:**
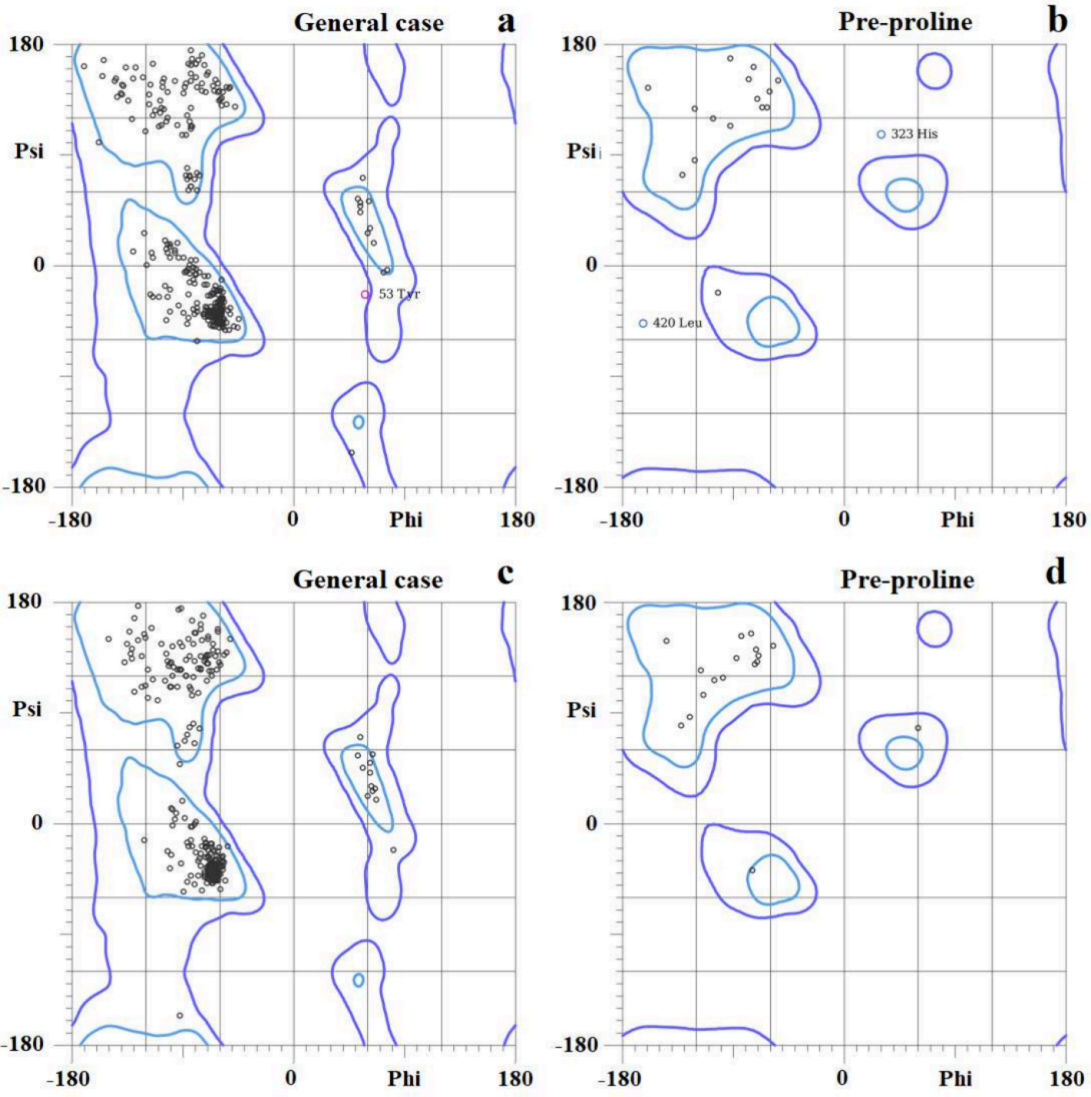
Ramachandran’s plot (MolProbity Server) shows the dihedral angles Φ and ψ of amino acid residues from GH_18_-cp*Leish*. The residues located in the most favored regions (98.4%) are shown in light blue curves, and the residues located in the additional allowed regions (100.0%) are shown in dark blue curves (1a and 1c). Ramachandran’s plot of GH_18_-cp*Leish* protein. 97.5% (421/432) of all residues were in favored (98%) regions. 99.3% (429/432) of all residues were in allowed (>99.8%) regions. There were 3 outliers (phi, psi): Tyr^53^ (58.6, −23.7). His^323^ (30.2, and 107.7). Leu^420^ (−164.9, −47.6). (1b and 1d). Ramachandran’s plot of GH_18_-cp*Leish* after being optimized by Coot software and energy minimized by Yasara software package. 98.4% (425/432) of all residues were in favored (98%) regions. 100.0% (432/432) of all residues were in allowed (>99.8%) regions. There were no outliers.

The GH_18_-cp*Leish* 3D model was constructed using the Robetta Server algorithm, and then, the quality of the predicted structure was checked in the MolProbity Server. It was observed that the vast majority of Φ (phi) – ψ (psi) dihedral angle pairs were distributed in the most favored and allowed regions of Ramachandran’s plot (Figure 1a,b), 97.5% (421/432) of all residues were in favored (98%) regions, and 99.3% (429/432) of all residues were in allowed (>99.8%) regions. There were only three outliers (Φ, ψ): Tyr^53^, His^323^, and Leu^420^ with corresponding coordinates (58.6, −23.7), (30.2, 107.7), and (−164.9, −47.6), respectively. To optimize the GH_18_-cp*Leish* 3D model, we used WinCoot software to reposition the amino acids Tyr^53^, His^323^, and Leu^420^ into the allowed regions of the Ramachandran plot (Figure 1c,d). The quality obtained for the GH_18_-cp*Leish* 3D model after being optimized is summarized in Table 2.

**Table 2:** MolProbity Result Summary and Criterion Chart for the GH_18_-cp*Leish* Structure^a^

parameters		scores/%		reference values
all-atom contacts	clash score, all atoms:	9.48		74th percentile* (*N* = 1784, all resolutions)
	clash score is the number of serious steric overlaps (>0.4 Å) per 1000 atoms.			
protein geometry	poor rotamers	1	0.29%	goal: <0.3%
	favored rotamers	346	99.14%	goal: >98%
	Ramachandran outliers	0	0.00%	goal: <0.05%
	Ramachandran favored	425	98.38%	goal: >98%
	Rama distribution Z-score	0.76 ± 0.37		goal: abs (Z score) < 2
	MolProbity score^c^	1.50		95th percentile^b^ (*N* = 27675, 0–99 Å)
peptide omegas	cis prolines:	0/21	0.00%	expected: ≤1 per chain, or ≤5%
low-resolution criteria	CA geometry outliers	2	0.47%	goal: <0.5%

a In the two-column results, the left column gives the raw count, right column gives the percentage.

b The 100th percentile is the best among structures of comparable resolution; the 0th percentile is the worst. For the clash score, the comparative set of structures was selected in 2004, and for MolProbity score in 2006.

c MolProbity score combines the clash score, rotamer, and Ramachandran evaluations into a single score, normalized to be on the same scale as X-ray resolution.

**Figure 2:**
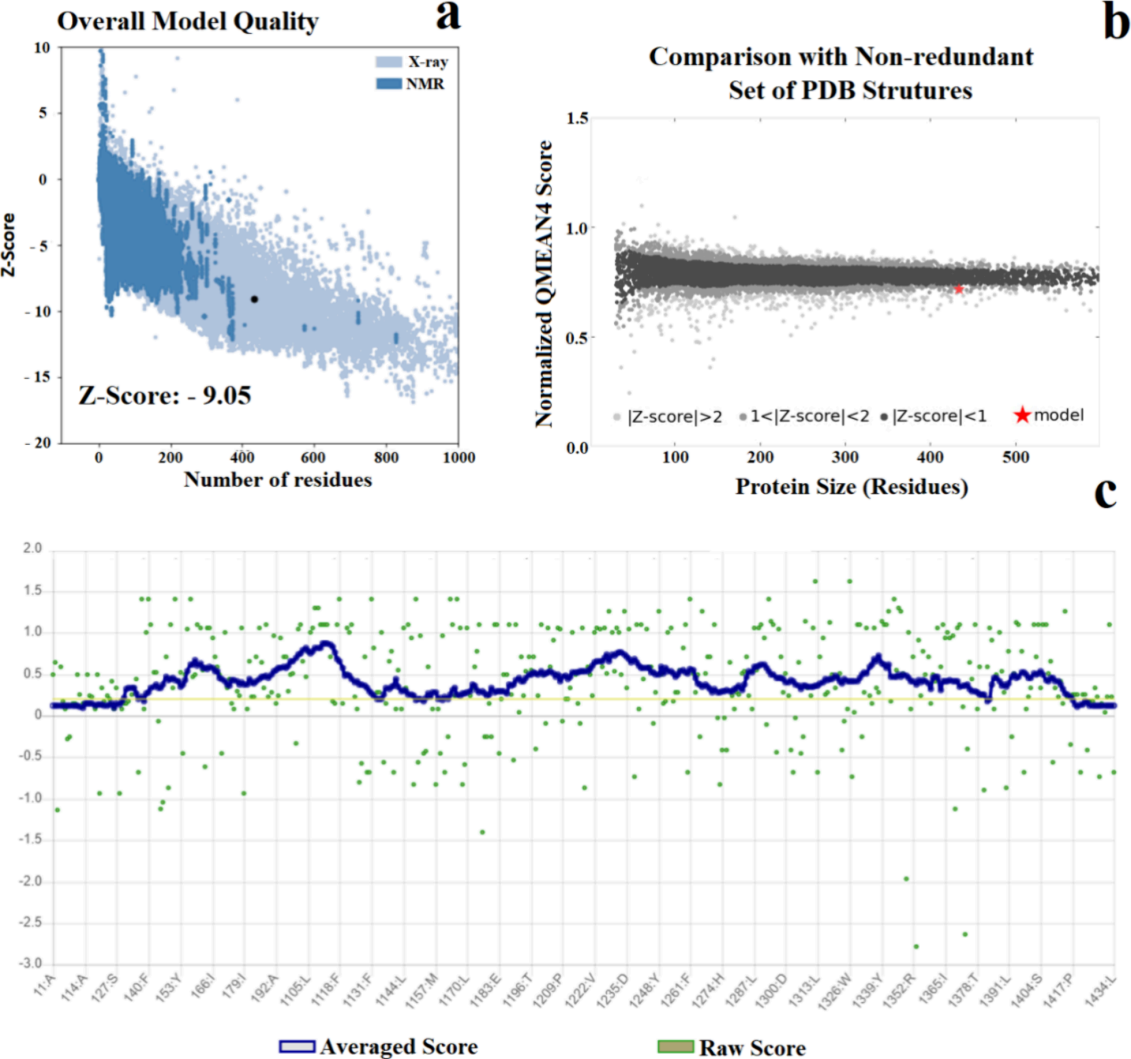
Validation of the in silico predicted model of GH_18_-cp*Leish* by ProSa-Web, QMEAN_4_, and VERIFY 3D. (2a) The *Z*-score (−9.05) of GH_18_-cp*Leish* falls in the range of values commonly found for PDB proteins whose structures were determined by NMR (dark blue region) and X-ray crystallography (light blue region). Note the black dot on the plot, which represents the *Z*-score of GH_18_-cp*Leish*. (2b). Structure validation by QMEAN, which shows the QMEAN-score of the predicted model of GH_18_-cp*Leish* (red star), when compared to a nonredundant set of high-resolution experimental structures (gray and black dots). (2c). Structure validation by VERIFY 3D, which shows the 3D-1D score for each atom of the predicted model of GH_18_-cp*Leish*. The graphic shows that 88.02% of the residues of the in silico structure of GH_18_-cp*Leish* presented a compatibility score of 0.2 or higher, which indicates that the structure is a high-quality model according to VERIFY 3D. Taken together these results suggest that the GH_18_-cp*Leish* model proposed here is of high quality and confidence.

Using the ProSA-Web Server, it obtained a value of −9.05 (black dot, Figure 2a). This result suggests that this model can be considered a good quality structure and is placed on a range of scores typically found for native conformations of proteins with similar sizes, around 400 amino acid residues, in which the three-dimensional structures were solved by X-ray diffraction. On the other hand, the results obtained by the QMEAN4 Server indicate that the structure of GH_18_-cp*Leish* presented a QMEAN-score of −1.40, comparable to that of high-resolution experimental structures (Figure 2b). Similarly, after using Verify 3D Server to assess the local quality, the GH_18_-cp*Leish* structure presented a total of 88.02% of residues that have averaged a 3D-1D score > =0.2 (Figure 2c), and at least 80% of the amino acids have scored > =0.2 in the 3*D*/1D profile.

**Figure 3:**
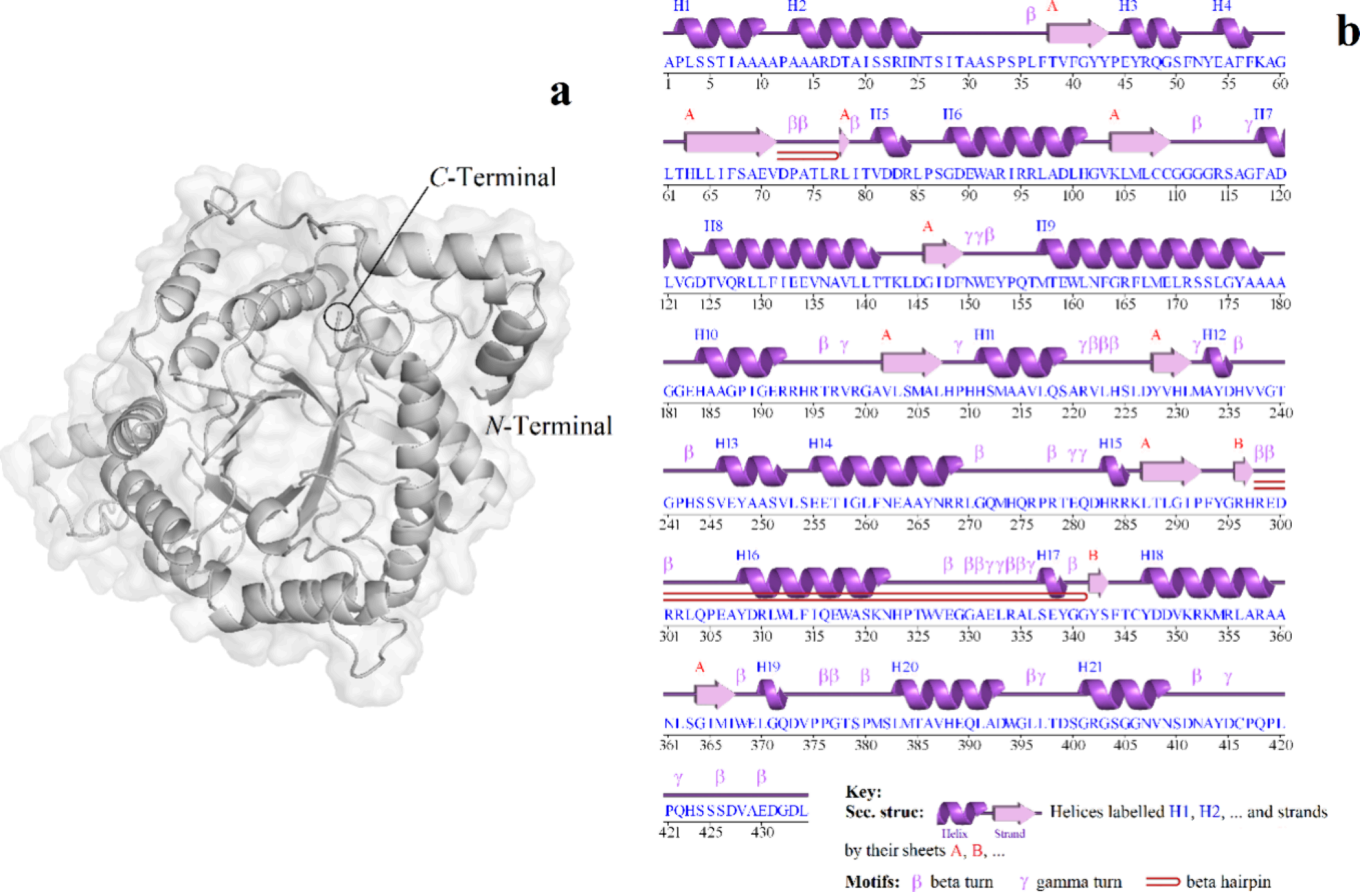
3D representation of the GH_18_-cp*Leish* protein with (a) cartoon and surface solvent mapping generated by Pymol software and (b) PDBsum 3D structure contents overview of the GH_18_-cp*Leish* showing all regions ordered in α-helixes, extended-strands, and random coils.

**Figure 4:**
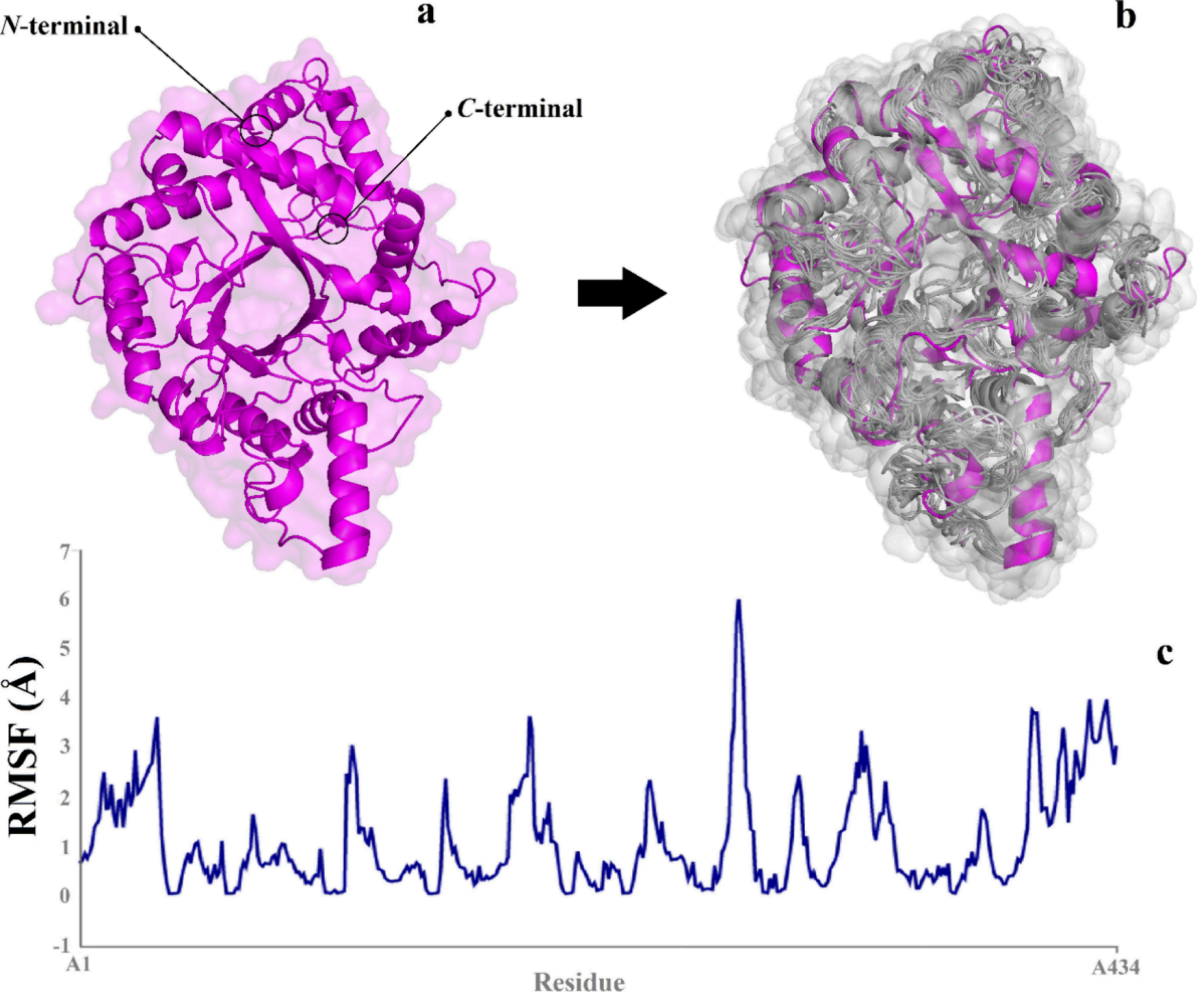
Molecular dynamics with CABS flex server 2.0 (http://biocomp.chem.uw.edu.pl/CABSflex2) representations for root mean square fluctuation (RMSF) based conformational coarse grain fluctuations for the flexibility and rigidity of the GH_18_-cp*Leish* chimeric protein. (a) 3D model of the GH_18_-cp*Leish*; (b) cartoon/surface representations of initial to final fluctuations; (c) RMSF vs residual plots with lower flexibilities at their bounded form of GH_18_-cp*Leish*.

**Figure 5:**
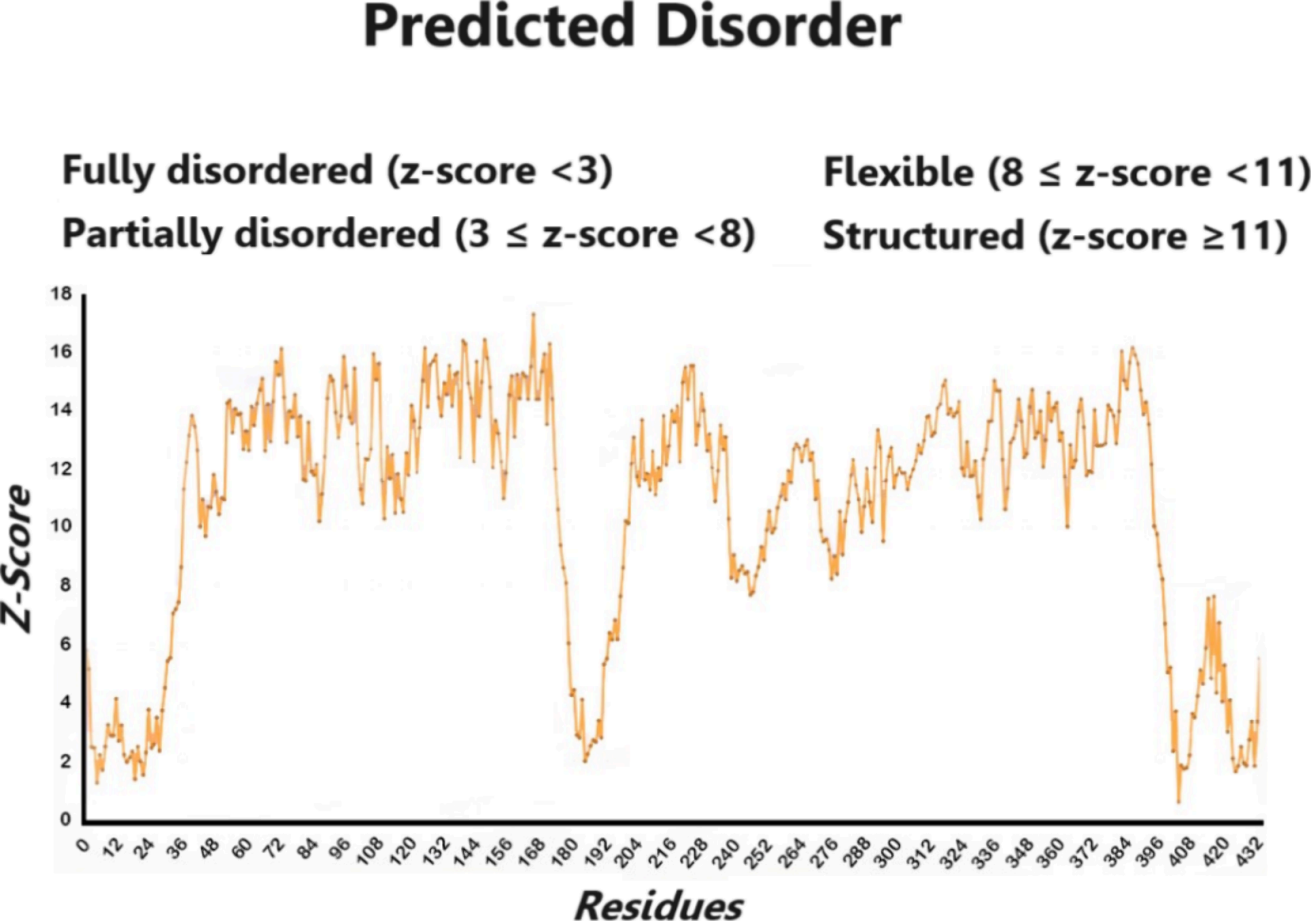
Residues with a wavy underline represent intrinsically disordered regions (IDR) predicted by the server ADOPT (Attention DisOrder PredicTor Server (https://adopt.peptone.io/)).

In addition, the TM-score and root-mean-square deviation (RMSD) obtained from the comparison between the raw model (GH_18_-cp*Leish*) and optimized and energy-minimized model showed a TM-score equal to 0.9967 (*d*_0_ = 7.48 Å) and an RMSD of 0.430 Å. Furthermore, the RMSDs obtained for the GH_18_ natives of *L. braziliensis*, *L. donovani*, *L. infantum*, *L. major*, *L. mexicana*, and *L. panamensis* indicate deviations (in Å) of 0.107, 0.056, 0.106, 0.101, 0.098, and 0.121, respectively, when compared to GH_18_-cp*Leish*. Additional analysis indicates that the mean percentage of Cα atoms (GH_18_-cp*Leish*) fitting under four distance thresholds, based on the GDT-TS score, is 0.9971% (*d* < 1 Å), 0.9885% (*d* < 2 Å), and 1.0000% (*d* < 4 Å). The GDT-HA score shows values of 0.9435% (*d* < 0.5 Å), 0.7857% (*d* < 1 Å), 0.9885% (*d* < 2 Å), and 1.0000% (*d* < 4 Å). Results obtained by MaxSub-score suggested value of 0.9855 (*d*_0_ = 3.50 Å). The final model is shown in Figure 3. Finally, the prediction of the protein structure disorder and fluctuation can be observed in Figures 4 and 5, respectively.

### Prediction of Antigenic, Allergenic, and Toxic Properties

The VaxiJen 2.0 Server performed immunogenicity prediction of the GH_18_-cp*Leish* (with no signal peptide) and showed a Score for the protective antigen = 0.5406, suggesting that this protein can operate as an antigen. Using the ABCpred Server to analyze the continuous B-cell epitopes, 42 peptides (16-*mers*) were identified in B-cell epitopes (Table S2). Still, out of these, only 20 peptides exhibited an ABCpred cutoff score ≥0.8 (Table 3).

**Table 3:** Continuous B-Cell Epitopes from Full Length (with No Signal Peptide) GH_18_-cp*Leish* Using the ABCpred Server

rank	peptide	score	mass (kDa)	pI	II	GRAVY
1	^402^RGSGGNVNSDNAYDCP^418^	0.95	16.25	4.21	33.31	–1.200
1	^148^DFNWEYPQTMTEWLNF^164^	0.95	21.21	3.57	17.09	–0.988
2	^364^GIMIWELGQDVPPGTS^380^	0.88	16.99	3.67	34.88	0.100
2	^343^SFTCYDDVKRKMRLAR^359^	0.88	19.89	9.78	58.51	–0.881
2	^268^RRLGQMHQRPRTEQDH^284^	0.88	20.45	11.52	124.41	–2.431
3	^317^EWASKNHPTWVEGGAE^333^	0.87	17.97	4.75	–23.51	–1.188
3	^274^HQRPRTEQDHRRKLTL^290^	0.87	20.71	11.54	83.31	–2.356
3	^236^HVVGTGPHSSVEYAAS^252^	0.87	15.97	5.98	20.29	–0.031
3	^190^GERRHRTRVRGAVLSM^206^	0.87	18.81	12.18	41.89	–0.975
4	^395^GLLTDSGRGSGGNVNS^411^	0.86	14.90	5.84	18.51	–0.519
4	^389^EQLADWGLLTDSGRGS^405^	0.86	17.04	4.03	16.36	–0.606
4	^230^HLMAYDHVVGTGPHSS^246^	0.86	17.07	6.25	28.01	–0.200
5	^4^SSTIAAAAPAAARDTA^19^	0.84	14.44	5.55	36.09	0.394
5	^18^TAISSRHNTSITAASP^34^	0.84	16.13	9.44	82.44	–0.231
6	^140^LTTKLDGIDFNWEYPQ^156^	0.83	19.40	4.03	7.32	–0.756
7	^418^QPLPQHSSSDVAEDGD^434^	0.8	16.81	3.84	81.60	–1.275
7	^37^FTVFGYYPEYRQGSFN^53^	0.8	19.75	6.00	31.82	–0.637
7	^354^MRLARAANLSGIMIWE^370^	0.8	18.32	9.35	–0.64	0.481
7	^309^DRLWLFIQEWASKNHP^324^	0.8	20.40	6.75	18.63	–0.819
7	^154^PQTMTEWLNFGRFLME^170^	0.8	20.00	4.53	20.58	–0.362

The observed molecular masses of these peptides vary from 14.44 to 21.21 kDa, considering only those with an ABCpred cutoff score ≥0.8. To determine these masses, the Protparam Server (https://web.expasy.org/protparam/) was utilized due to Xtalpred-RT’s limitation in calculating small peptides (<30 amino acids). Among the 20 peptides exhibiting an ABCpred cutoff score ≥0.8, their molecular masses are distributed within the range of 14.44–21.21 kDa (Table S2). Regarding the pI values observed for these peptides, it was identified that 70% can function in acidic environments (pH: 3.57–6.75). In contrast, only six peptides operate in basic pH conditions (9.35–12.18). An analysis of the instability index revealed that 70% of the peptides are considered stable. Additionally, predictions of hydropathicity indicate that 85% of the identified peptides are hydrophilic.

The analysis of discontinuous B-cell epitopes using the analysis of helper T-cell epElliPro Server identified 26 epitopes (Table S3) ranging from 3 to 29 residues. Similar to the study of the continuous B-cell epitopes, we present only those with a cutoff score ≥0.8 (Table 4). Prediction of the IC_50_ values of the peptides (from GH_18_-cp*Leish*) suggesting the ability to interact and bind to the MHC class I and II molecules was performed by the IEDB online tool. This study identified cytotoxic T-cell epitopes (a total of 536 peptides) with a probability of high (12.50%), intermediate (23.88%), and low (63.62%) binding affinity. On the other hand, the analysis of helper T-cell epitopes showed a total of 175 peptides with a probability of high (78.46%) and intermediate (21.54%) binding affinity. The peptide with the highest affinity for T-cell epitopes according to each allele can be found in Table 5 (MHC-I) and Table 6 (MHC-II), respectively.

**Table 4:** Discontinuous B-Cell Epitopes from the Full Length (with No Signal Peptide) GH_18_-cp*Leish* Using the ElliPro Server

rank	residues (amino acids)	score
1	H^423^, S^424^, S^425^, S^426^, D^427^	0.99
2	A^319^, S^320^, H^323^	0.959
3	W^318^, K^321^, N^322^	0.957
4	V^428^, A^429^, E^430^, D^431^, G^432^, D^433^	0.944
5	N^267^, L^270^, G^271^, Q^272^, M^273^	0.936
6	S^410^, D^411^, N^412^, A^413^	0.893
7	R^27^6, P^277^, E^280^	0.869
8	P^419^, L^420^, P^421^	0.86
9	D^415^, C^416^, P^417^, Q^418^	0.851
10	A^1^, P^2^, L^3^, S^4^, S^5^, T^6^, I^7^, A^8^, A^9^, A^10^, A^11^, P^12^, A^13^, A^14^, A^15^, R^16^, D^17^, T^18^, A^19^, I^20^, S^21^, S^22^, R^23^, H^184^, A^185^, A^186^, G^187^, P^188^, I^189^	0.811

**Table 5:** Prediction of MHC-I Binding Epitopes

allele	length	peptide	IC_50_ (nM)^a^
HLA-A*02:03	9	^166^FLMELRSSL^174^	2.78
HLA-A*68:02	9	^256^ETIGLFNEA^264^	2.98
HLA-A*02:06	9	^221^RVLHSLDYV^229^	3.68
HLA-A*30:01	9	^197^RVRGAVLSM^205^	3.78
HLA-A*68:01	9	^157^MTEWLNFGR^165^	4.61
HLA-A*02:01	9	^166^FLMELRSSL^174^	5.52
HLA-A*11:01	10	^49^GSFNYEAFFK^58^	6.93
HLA-A*31:01	9	^157^MTEWLNFGR^165^	10.14
HLA-A*26:01	10	^37^FTVFGYYPEY^46^	17.26
HLA-A*30:02	10	^34^SPLFTVFGYY^43^	17.48
HLA-A*33:01	10	^162^NFGRFLMELR^171^	17.83
HLA-A*03:01	10	^49^GSFNYEAFFK^58^	24.56
HLA-A*24:02	10	^42^YYPEYRQGSF^51^	26.93
HLA-A*32:01	9	^310^RLWLFIQEW^318^	33.95
HLA-A*23:01	10	^42^YYPEYRQGSF^51^	37.59
HLA-A*01:01	10	^167^LMELRSSLGY^176^	41.7
			
HLA-B*08:01	10	^91^WARIRRLADL^100^	3.89
HLA-B*07:02	9	^379^SPMSLMTAV^387^	4.66
HLA-B*40:01	9	^132^IEEVNAVLL^140^	7.42
HLA-B*58:01	9	^318^WASKNHPTW^326^	9.22
HLA-B*35:01	9	^32^SPSPLFTVF^40^	14.65
HLA-B*44:03	9	^168^MELRSSLGY^176^	15.44
HLA-B*15:01	10	^31^ASPSPLFTVF^40^	15.84
HLA-B*44:02	9	^168^MELRSSLGY^176^	23.35
HLA-B*57:01	9	^318^WASKNHPTW^326^	60.18
HLA-B*51:01	9	^379^SPMSLMTAV^387^	992.97

a IC_50_(nM) = The predicted output is given in units of IC_50_ nM. A lower number indicates higher affinity. Peptides with IC_50_ values <50 nM are considered high affinity <500 nM intermediate affinity and <5000 nM low affinity. Most known epitopes have a high or intermediate affinity. Some epitopes have a low affinity, but no known T-cell epitope has an IC_50_ value greater than 5000. All alleles highlighted in bold are among the top 10 HLA-A and HLA-B alleles with the highest frequencies in the South and Central American regions, based on the Allele Frequency Net Database (https://www.allelefrequencies.net/hla.asp).

**Table 6:** Prediction of MHC-II Binding Epitopes

allele	length	peptide	percentile rank^a^
HLA-DRB1*03:01	12	^76^LRLITVDDRLPSGDEW^91^	0.49
HLA-DRB1*07:01		^213^MAAVLQSARVLHSLDY^228^	8.7
HLA-DRB1*15:01		^381^MSLMTAVHEQLADWGL^396^	20
HLA-DRB3*01:01		^129^LLFIEEVNAVLLTTKL^144^	31
HLA-DRB4*01:01		^46^YRQGSFNYEAFFKAGL^61^	38
HLA-DRB5*01:01		^259^GLFNEAAYNRRLGQMH^274^	51

a Percentile rank = IC_50_ value; low percentile rank = high-level binding. A lower number indicates higher affinity. Peptides with IC_50_ values <50 nM are considered high affinity <500 nM intermediate affinity and <5000 nM low affinity. All alleles highlighted in bold are among the top 10 HLA-DRB alleles with the highest frequencies in the South and Central American regions, based on the Allele Frequency Net Database (https://www.allelefrequencies.net/hla.asp).

**Figure 6:**
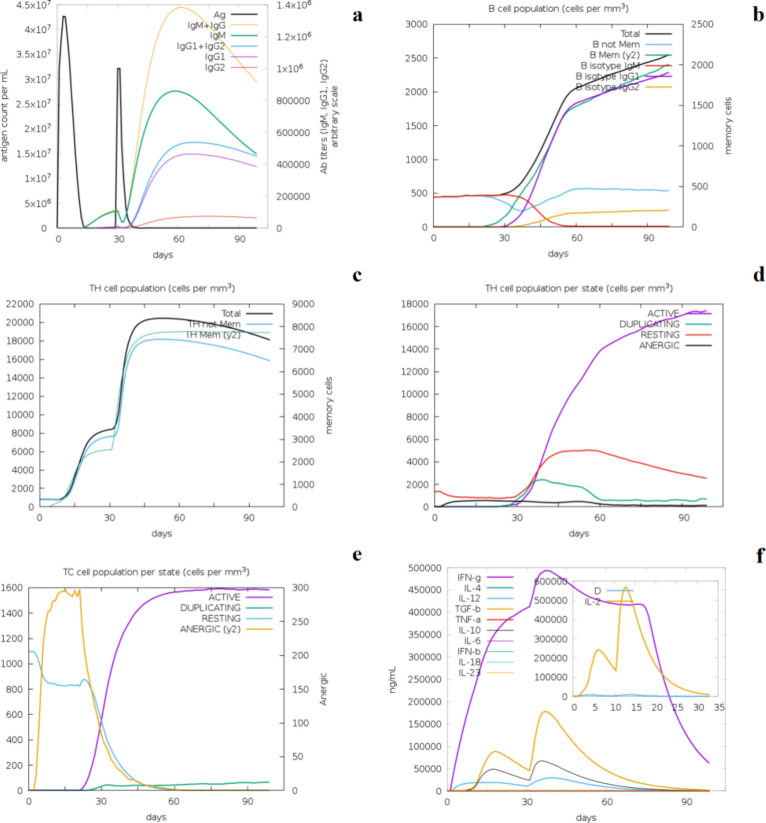
Prediction of the immune simulation with vaccine construct with (a) Generation of antibodies after administration of two vaccine doses. Different isotypes and combinations of antibodies are shown as colored lines, and antigens are shown as black lines. (b) Total B-cell response after vaccine doses shown in black line and colored lines denoting various B isoforms cells. (c) Proliferation of total T-Helper cells is shown in the black line and the T-Helper cell memory curve is shown in the blue line. (d) Active and resting T-Helper cell population after vaccines indicated by magenta and red lines denoting, respectively. Resting T-cells are those that have not encountered the antigen, and anergic cells (black line) signify the T-cells which are tolerant to antigens. (e) Active and resting Cytotoxic T-cell proliferation after vaccine dose. (f) The various cytokine profiles are shown as colored peaks with prominent up-regulation of proinflammatory IFN-γ (magenta line). The inserted graph shows Simpson index D of IL-2 where D is a measure of diversity.

In silico humoral and cell-mediated immune responses were also predicted by the simulations with the C-ImmSim web Server. After the first dose, antigen counts reached approximately 4.3 × 10^7^/mL, with the maximum peak on the fifth day, dipping to 0 on the 12th day. A markedly basal level of IgM + IgG was noted until the 12th day, and then, the population started to grow exponentially; however, it remained below 4.5 × 10^7^/mL. After administering the second dose, the antigen counts quickly reached approximately 3.3 × 10^7^/mL and lasted approximately 1 week. IgM + IgG and IgG_1_ + IgG_2_ reached their maximum peaks of 4.5 × 10^7^/mL and 1.7 × 10^7^/mL around the 55th day, after which the indices began to decline over time (measured in days) (Figure 6a). The data obtained show that total B cell counts did not increase during the first dose of the vaccine and remained below 500 cells/mm^3^. However, after the second dose was administered, total B cell levels were shown to increase. The total number of B cells in the cell population continued to grow until after day 90 (2500 cells/mm^3^) (Figure 6b), indicating that this cell group needs at least two doses to respond against GH_18_-cp*Leish*. Levels of upregulated memory B cells, IgG_1_ B subtype, and IgG_2_ B subtype persisted over 90 days, representing the entire simulation period. Cell-mediated immunity was activated, as indicated by increased numbers of active CTLs, HTLs, and memory helper T cells. A clear indication of memory helper T cell proliferation starting from the second dose is shown in Figure 6c. The active T helper cell population increased to 5,800 cells/mm^3^ on day 30 after the first dose and peaked (16,500 cells/mm3) on day 50 after the second dose (Figure 6d).

The active cytotoxic T-lymphocytes attained a population with a peak at around 600 cells/mm^3^ after the first dose of the vaccine. After the second administration, the cytotoxic T-lymphocyte population continued to grow, which remained stable until the 70th day (Figure 6e). The number of resting cytotoxic T-cell populations dropped vigorously and virtually disappeared by the 30th day after the second dose of the vaccine. Lastly, the level of proinflammatory cytokine IFN-γ (5 × 10^5^ molecules/mm^3^) was hugely amplified, but in contrast to that, a shallow level of anti-inflammatory cytokine IL-10 was noted (Figure 6f). Finally, the allergenicity and toxicity potential analysis performed by AlgPred, AllergenFP, and ToxinPred servers found no evidence of allergenicity or toxicity for GH_18_-cp*Leish*.

### TLRs Receptor and GH_18_-cp*Leish* Peptide Molecular Docking and Molecular Dynamics Studies

**Figure 7:**
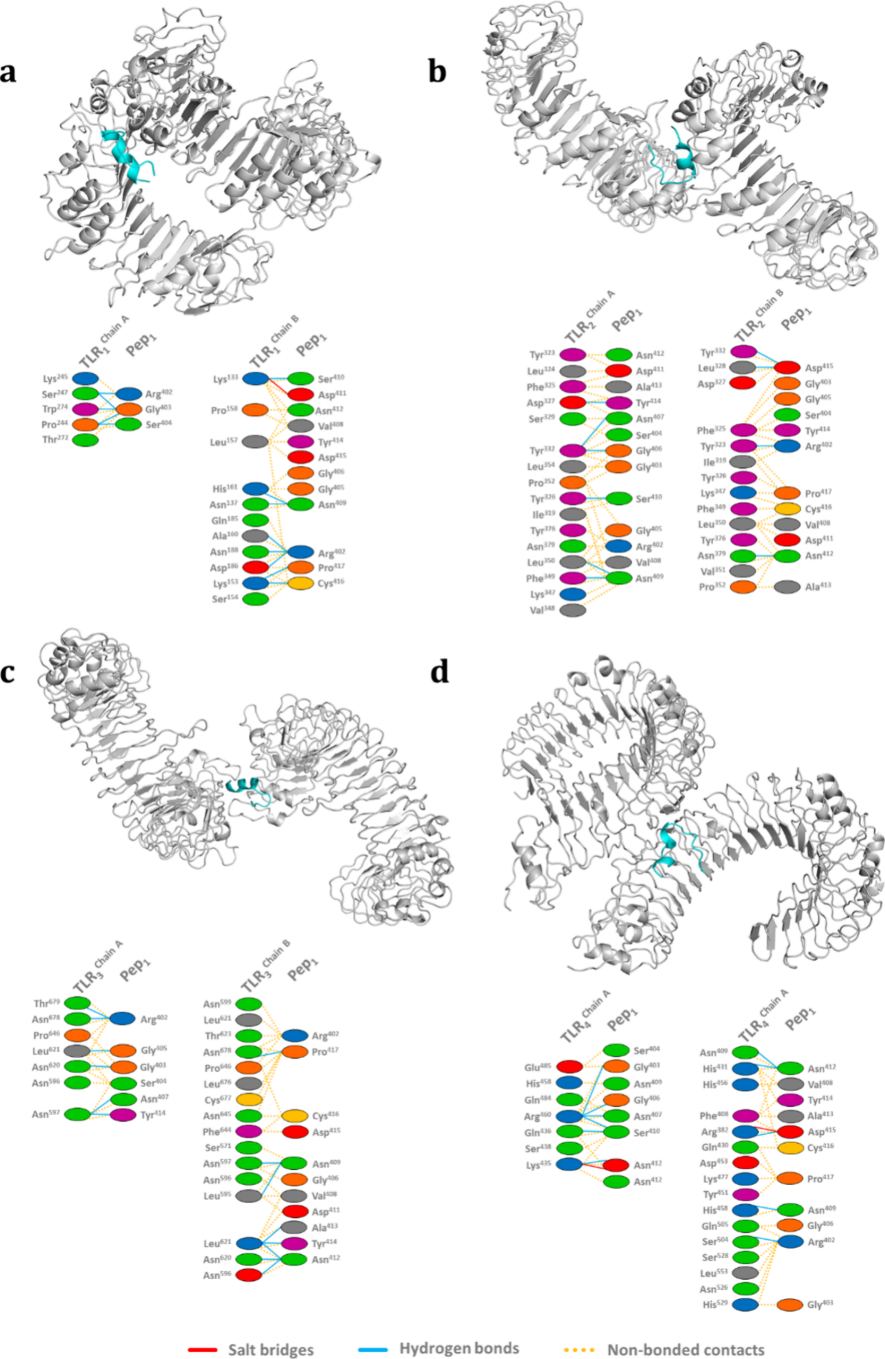
Prediction of the binding mode of different human TLRs and Peptide 1 (Pep_1_-cp*Leish*). (a) Mode of interaction between TLR_1_ (PDB ID: 6NIH) and Pep_1_-cp*Leish*. (b) Mode of interaction between TLR_2_ (PDB ID: 6NIG) and Pep_1_-cp*Leish*. (c) Mode of interaction between TLR_3_ (PDB ID: 7WVJ) and Pep_1_-cp*Leish*. (d) Mode of interaction between TLR_4_ (PDB ID: 3FXI) and Pep_1_-cp*Leish*. All predictions were conducted using the ClusPro 2.0 Protein–protein Docking server (https://cluspro.org/login.php), and figures were generated using PDBsum (https://www.ebi.ac.uk/thornton-srv/databases/pdbsum/Generate.html). All positive residues (His, Lys, and Arg) are indicated in blue; negative residues (Asp and Glu) in red; neutral residues (Ser, Thr, Asn, and Gln) in green; aliphatic residues (Ala, Val, Leu, Ile, and Met) in gray; aromatic residues (Phe, Tyr, and Trp) in magenta; residues Pro and Gly are represented in orange, while Cys is in yellow.

The prediction of interactions and binding affinity was performed between peptide 1, which is the one with the highest score (obtained from GH_18_-cp*Leish*) based on the ABCpred Server score, and the receptors TLR_1_, TLR_2_, TLR_3_, and TLR_4_ (Figure 7). All simulations were carried out with the help of the ClusPro server. A total of 21, 11, 19, and 27 simulations were performed for the complexes Pep_1_-cp*Leish*::TLR_1_ (Figure 7a), Pep_1_-cp*Leish*::TLR_2_ (Figure 7b), Pep_1_-cp*Leish*::TLR_3_ (Figure 7c), and Pep_1_-cp*Leish*::TLR_4_ (Figure 7d), respectively. The best interaction cluster score was observed to complex Pep_1_-cp*Leish*::TLR_2_ (Center = −622.6 and Lowest Energy = −841.7 kcal.mol^–1^) following by the complexes Pep_1_-cp*Leish*::TLR_4_ (Center = −590.3 and Lowest Energy = −590.3 kcal.mol^–1^), Pep_1_-cp*Leish*::TLR_3_ (Center = −589.1 and Lowest Energy = −657.0 kcal.mol^–1^), and Pep_1_-cp*Leish*::TLR_1_ (Center = −504.1 and Lowest Energy = −602.9 kcal.mol^–1^).

The results of the docking simulations show that the Pep_1_-cp*Leish*::TLRs complexes encompass a network characterized by aromatic, cation-π, hydrophobic, ionic, and sulfur interactions and hydrogen bonds. Regarding aromatic, cation-π, and sulfur interactions, they were observed only in the Pep_1_-cp*Leish*::TLR_2_ complex, with 2, 2, and 1 occurrences, respectively. Ionic interactions were found in all complexes except the Pep_1_-cp*Leish*::TLR_2_ complex. 2, 2, and 4 interactions were observed in the Pep_1_-cp*Leish*::TLR_1_, Pep_1_-cp*Leish*::TLR_3_, and Pep_1_-cp*Leish*::TLR_4_ complexes, respectively. Hydrophobic interactions were found in all complexes, with 3 in Pep_1_-cp*Leish*::TLR_1_, 12 in Pep_1_-cp*Leish*::TLR_2_, 3 in Pep_1_-cp*Leish*::TLR_3_, and 1 in Pep_1_-cp*Leish*::TLR_4_ complexes. Similar hydrogen bonds were shown in all complexes. Our results suggest the existence of 42 bonds in both complexes, Pep_1_-cp*Leish*::TLR_2_ and Pep_1_-cp*Leish*::TLR_4_, while 43 bonds were observed in both the Pep_1_-cp*Leish*::TLR_1_ and Pep_1_-cp*Leish*::TLR_3_ complexes. In all complexes, the three types of hydrogen bonds were observed: Main Chain::Main Chain (3, 1, 3, and 1), Main Chain::Side Chain (9, 21, 9, and 21), and Side Chain::Side Chain (31, 20, 31, and 20) in the Pep_1_-cp*Leish*::TLR_1_ - Pep_1_-cp*Leish*::TLR_4_ complexes, respectively (Table S4).

Using the PRODIGY Server, Δ*G* values were obtained for the Pep_1_-cp*Leish*::TLR_1_ - Pep_1_-cp*Leish*::TLR_4_ complexes, resulting in −14.6, −19.9, −13.3, and −12.8 kcal.mol^–1^, along with dissociation constant (*K*_d_(M)) values of 1.9 × 10^–11^, 2.4 × 10^–15^, 1.8 × 10^–10^, and 3.8 × 10^–10^, respectively. These values indicate that such interactions are energetically favorable.

Since the interactions between TLRs and the Pep_1_-cp*Leish* peptide are dynamic processes, MD simulations were performed to better understand the mode of interaction between them. TLR_1_ (the highest binding energy, which corresponds to the least favorable interaction) was selected to demonstrate the general behavior of these complexes. MD simulations conducted in the presence of Pep_1_-cp*Leish* indicated that the Pep_1_-cp*Leish*::TLR_1_ complex exhibited stable behavior at the predicted interaction site, as observed in its RMSD (Figure S7b). However, the structure of Pep_1_-cp*Leish* underwent significant rearrangement throughout the simulation (Figure S7a) compared with the molecular docking data. It is possible that this new, more linear structure allows Pep_1_-cp*Leish* to assume greater stability when interacting with TLR_1_, as shown in Video S1. Interestingly, the MD simulations indicated that the last four residues (^13^YDCP^16^) in the *C*-terminal region contributed to a disorder in accommodation, suggesting the potential for future improvements that could be achieved by shortening Pep_1_-cp*Leish*. Despite this, Pep_1_-cp*Leish* stabilization was evident based on both the docking and MD results, particularly in the *N*-terminal region. This structural rearrangement, particularly in the *N*-terminal region, may influence and demonstrate the effectiveness of Pep_1_-cp*Leish*’s interaction with TLRs, as shown with TLR_1_.

The variation in the radius of gyration (*R*_g_) as a function of simulation time suggests that the stabilization of the Pep_1_-cp*Leish*::TLR_1_ complex during the MD simulation led to a relatively compact state. This indicates that the TLR_1_ likely adjusted its folding to accommodate Pep_1_-cp*Leish*, suggesting a possible state of structural equilibrium. Additionally, the fluctuations observed between 70 and 90 ns (Figure S7c) represent a region of significant variability. These fluctuations may indicate temporary instability in the interaction between Pep_1_-cp*Leish* and TLR_1_ or structural rearrangements within the complex. This dynamic behavior could be associated with the reorganization of side chains to optimize intermolecular interactions or local changes in TLR_1_ caused by the docking or movement of Pep_1_-cp*Leish* at the interaction site. However, a return to typical fluctuations and a more stable profile is observed before the simulation reaches 100 ns, reinforcing the system’s ability to restabilize. This suggests that the rearrangements occurring between 70 and 90 ns were transient and likely necessary to achieve a more energetically favorable state.

The root-mean-square fluctuation (RMSF), which measures the fluctuation of individual atoms around their average positions, was calculated for all Cα atoms of TLR_1_ over the 100 ns simulation (Figure S7d). Most residues of TLR_1_ (Chain A) exhibited fluctuations with an intensity of less than 0.3 nm, while most residues of chain B exceeded 0.3 nm in fluctuation. In both TLR_1_ chains, some residues exhibited fluctuations exceeding 0.5 nm, particularly those in the *C*-terminal regions of chain A. Additional analysis revealed that the fluctuations of residues involved in interactions with Pep_1_-cp*Leish* did not exceed 0.3 nm. Regarding hydrogen bonding (H-bonds), six bonds were present for most of the simulation time (100 ns). However, nine H-bonds were observed at 8 ns, and no H-bonds were observed near 90 ns (Figure S7e).

Lennard–Jones interaction analysis indicates the presence of 56 interactions in the Pep_1_-cp*Leish*::TLR_1_ complex. Among these, 11 were identified as van der Waals (VdW) interactions, which act on short scales and are not charge-dependent; 22 were van der Waals clash interactions, indicative of excessive atomic overlap resulting in strong repulsion represented by the r^–12^ term; and 23 proximal interactions were identified, associated with close contacts between atoms or molecular groups that lack a highly specific interaction type (Table S5). Overall, the Lennard–Jones interactions correspond to dispersion and repulsion forces related to the spatial organization and proximity of the atoms. Electrostatic interaction analysis revealed 25 interactions, distributed as follows: 15 hydrogen bonds, of which 10 represent weak hydrogen bonds with a strong electrostatic contribution (resulting from dipole interactions) and partial stabilization by van der Waals dispersion forces; 1 ionic interaction, which is purely electrostatic and represents forces between opposite charges; 8 polar contacts (including weak ones), reflecting electrostatic interactions between polar groups; and 1 carbonyl interaction, classified as a polar interaction typically associated with electrostatic forces (Table S5). Together, these electrostatic interactions involve forces between the full or partial charges of polar or ionic molecules.

These results suggest that GH_18_-cp*Leish* may be a potential source of peptides capable of inducing anti-*Leishmania* defense responses.

## Discussion

The availability of complete genomes of parasites of clinical interest has enabled different research groups to employ computational approaches to develop safer vaccines. Although the design of the antimeningitis vaccine^57,58^ was the precursor to the technique of reverse vaccinology, it opened up countless possibilities for new rational vaccine designs.

Omics-guided approaches, including proteomics, transcriptomics, and genomics, have been effectively employed, resulting in vaccine designs against different pathogens of clinical interest in humans and other animals. Among the vaccines developed using these techniques are those directed to the West Nile virus^59^ and SARS-Cov-2,^60^ to bacteria, including the species *Acinetobacter baumannii*,^61^*Bacillus anthracis*,^62^*Bordetella pertussis*,^63^*Chlamydia pneumoniae*,^64^*Escherichia coli*,^37,65^*Neisseria gonorrheae*,^66^*Salmonella typhimurium*,^67^*Staphylococcus aureus*,^36,68^*Streptococcus pneumonia*,^69^ and *Rickettsia prowazekii*.^70^ Vaccines have also been reported as ways to control infections caused by fungi and protozoa such as *Histoplasma capsulatum*,^71^*Cryptosporidium hominis*,^40^*Plasmodium falciparum*,^72^*Schistosoma hematobium*,^73^*Theileria annulata*,^74^*Toxoplasma gondii*,^75^ and *Trypanosoma brucei* gambiense.^76^ A few anti-*Leishmania* vaccines have been developed against various species of *Leishmania*, including *L. donovani*,^77^*L. infantum*,^78^*L. major*,^79^*L. martiniquensis,* and *L. orientalis*.^80^ There is currently no vaccine that is accessible for clinical or commercial use in humans.

To minimize this public health problem, we employed all available *Leishmania* spp. genomes in biological databases as bait (accession: lbz: LBRM_16_0800) to construct a chimeric chitinase, we call ourselves GH_18_-cp*Leish*. Subsequently, we utilized immunoinformatics and bioinformatics tools to forecast potential peptides that could induce immunogenic responses against *Leishmania* spp. In silico approaches provide cost-effective and less harmful alternatives to traditional vaccine production by reducing the need for extensive time and laboratory experiments.^63^ Typically, vaccines are designed to induce antibody-mediated immunity (AMI) through B-cells. Based on this principle, our anti-*Leishmania* vaccine design also utilized this method due to the demonstrated immediate and efficient immune responses, particularly in immunologically immature populations.

Conversely, T-cells can confer long-lasting immunity compared to antibody-mediated immunity, as the latter can be easily overcome by antigenic drift.^81^ Therefore, we propose using peptides obtained from GH_18_-cp*Leish* in experimental *in vitro* and *in vivo* models to evaluate their ability to elicit both humoral- and/or cell-mediated immune responses.

To optimize the efficiency of the proposed anti-*Leishmania* vaccine, we carefully engineered the gene encoding the GH_18_-cp*Leish* protein to exhibit optimal sequence characteristics for expression in *K. pastoris*, as shown in Figures S1 and S2. Secondary structure analysis of the mRNA sequence suggests that the MFE structure (free energy: Δ*G* = −381.70 kcal.mol^–1^), depicted in Figure S2a, naturally folds into a thermodynamically stable conformation. The colors indicate the probability of base pairing, ranging from 0 (blue) to 1 (red).^82,83^ Conversely, the centroid structure (Figure S2b) reflects an average of various possible conformations for the mRNA molecule.^84^ Its energy value (Δ*G* = −320.26 kcal.mol^–1^) is higher compared to that of the MFE, suggesting that, while less stable, it represents the range of conformations the RNA can adopt. The Mountain Plot (Figure S2c) shows that the MFE (red) and centroid (blue) structures share some similarities but differ in specific regions of the sequence. The green line (pf) marks the regions with higher structural stability, where bases have a greater probability of being paired in all possible conformations.^82,83^ The pf index, which approximates either the red or blue curve, indicates that both the MFE and centroid structures align with the most probable pairing regions. Consequently, the mRNA encoding GH_18_-cp*Leish* is more likely to assume the MFE structure, making it a more stable conformation. Entropy analysis (Figure S2d) reveals conformational diversity at each position along the mRNA sequence. Higher entropy values indicate greater conformational variability, while lower values correspond to more stable regions, suggesting that certain areas of the structure are less prone to conformational changes.

Additionally, the CAI (codon adaptation index) value of 1 for the GH_18_-cp*Leish* coding mRNA suggests that the sequence is optimized for efficient translation in the specific host system.^85^ As reported elsewhere, the CAI is not directly related to the mRNA structure but instead reflects the efficiency of the mRNA translation process.^86^ Therefore, an optimal CAI does not necessarily guarantee the desired structural conformation.

The expression of recombinant proteins intended for clinical application in yeast and other systems has not only resulted in increased productivity and yield but also in efficiency and precision.^87^ The primary sequence of GH_18_-cp*Leish*, like other GH_18_ chitinases, exhibits specific patterns, including total length and the presence of a signal peptide (Table 1). Additionally, physicochemical properties such as theoretical molecular weight (*Mr*), isoelectric point (pI), aliphatic index (AI), hydrophobicity index (GRAVY), domains, and disulfide bonds are shared between GH_18_-cp*Leish* and other GH_18_ proteins.^88−93^

The structural analysis of GH_18_-cp*Leish* reveals canonical characteristics similar to other GH_18_ proteins, including known features contributing to forming the catalytic site at the carboxyl end of the β-barrel. Holistically, the overall structure shows (β/α)_8_ regions, which together form a TIM-barrel protein fold.^94,95^ The GH_18_-cp*Leish* has a core region comprising eight parallel β-strands, where its catalytic site is located. This site consists of the highly conserved Asp^145^-X-X-Asp^148^-X-Asp^150^-X-Glu^152^ motif responsible for the catalytic mechanism involving hydrolysis. In this motif, ‘X’ represents any of the other known amino acid residues (Figure S1a,b).

All of the results obtained from the analysis of the GH_18_-cp*Leish* model, including measurements of the overall model quality (Z-score), overall quality factor, 3D-1D averaged score, and stereochemical quality (Figures 1–5), suggest that the design of the chimeric protein exhibits physical and chemical characteristics compatible with proteins in GH_18_. Thus, the structure of GH_18_-cp*Leish* can be classified as a good-quality model, falling within the range of scores commonly observed in native protein conformations of similar sizes and structures solved by X-ray diffraction, as reported in previous publications.^96^

The VaxiJen 2.0 server predicted GH_18_-cp*Leish* as a potential activator of the host immune system based on a score of 0.5406. The server follows a two-stage process: (I) it identifies antigenic peptides, and then (II) it identifies peptides with the highest antigenicity scores.^31,33^ Scores similar to those found in this work have been previously demonstrated in other publications.^65,97^ We also identified continuous (Table 3) and discontinuous (Table 4) epitopes that can efficiently interact with B cells and serve as triggers for the immunologic system. The recognition of epitopes by B cells occurs due to the accessibility of pockets or cavities present in the surface receptors of specific lymphocytes as well as the flexibility and hydrophilicity of the elicitor. Both continuous and discontinuous epitope predictions are based on an artificial neural network designed to discover linear (peptides) and discontinuous (unique amino acids) B-cell epitope regions within an antigen (protein) sequence.^98,99^ Typically, the B-cell epitopes identified constitute valuable peptides for synthetic vaccine candidates, disease diagnosis, and allergy research.^37^

This search also revealed several high-priority T-cell-eliciting epitopes, as shown in Tables 5 and 6. High-priority epitopes possess the ability to bind tightly to T-cell receptors, thereby triggering a more effective immune response. Similar to B-cell epitopes, these T-cell epitopes are of particular interest in biomedical research and vaccine development. Although T-cell epitopes offer the advantage of stimulating the immune system against specific targets.^100^ Similarly, the prediction of T cell elicitor epitopes makes use of neural networks to predict the affinity or binding capacity of specific epitopes to MHC type I or II molecules.^101,102^ Epitopes recognized by MHC are more likely to be effectively presented to T cells. The predicted peptides showed comprehensive population coverage, with a particular focus on Brazilian ethnic groups, where leishmaniasis poses a significant public health challenge.^103,104^

Thus, the rational selection of immunogenic epitopes from specific proteins like GH_18_-cp*Leish*, based on predictions of the most immunogenic regions, can be an effective strategy.^105,106^ The peptide Pep_1_-cp*Leish* (^402^RGSGGNVNSDNAYDCP^418^) was identified and selected as highly immunogenic through various epitope prediction models, including MHC-I and MHC-II binding predictions, immunogenicity prediction tools such as IEDB, and molecular docking and dynamics simulations. Focusing on Pep_1_-cp*Leish* simplifies vaccine development by minimizing the inclusion of specific GH_18_-cp*Leish* regions that do not contribute to immunogenicity or may induce tolerance or undesirable responses. This approach also reduces the risk of cross-reactions, which are more likely when using the complete protein, as it may provoke immune responses against native host proteins.^107,108^ Furthermore, the selection of Pep_1_-cp*Leish*, rather than the entire GH_18_-cp*Leish* protein, is justified, because this epitope represents the immunogenic potential of the whole protein. While GH_18_-cp*Leish* may be broadly immunogenic, Pep_1_-cp*Leish* represents the critical region responsible for inducing the desired immune response.

The results of the computational docking and dynamics studies, using the peptide with the highest score obtained from the ABCperd Server (Pep_1_-cp*Leish*, with a value of 0.95), also suggest that several peptides identified in this work can efficiently bind to the TLRs (Figures 7, S3, Tables S4 and S5). As previously reported, GH_18_ chitinases are potent immune response inducers.^18−20^ In addition, the peptides present in the GH_18_-cp*Leish* seem capable of inducing an immune response based on helper T-lymphocyte (HTL) and cytotoxic T-lymphocyte (CTL) epitopes. HTL epitopes are necessary to induce high titers of proinflammatory cytokines during immune responses to infectious agents. Proinflammatory cytokines such as IFN-γ, IL-2, and TNF-α^109^ are essential for inducing T-cell activation and differentiation, promoting B-cell activation to synthesize and secrete antibodies, and triggering cellular immune responses mediated by macrophages, ultimately leading to the elimination of intracellular *Leishmania* spp. On the other hand, CTLs play a crucial role in low-dose parasite infections, as they help potentiate molecular mechanisms associated with secondary immune responses.

Analysis using the AlgPred, AllergenFP, and ToxinPred servers showed no evidence of allergenicity or toxicity for GH_18_-cp*Leish* or its peptides, corroborating the hypothesis that this protein is safe and a promising candidate for an anti-*Leishmania* vaccine. In addition, the proposed anti-*Leishmania* vaccine candidate, compared to other designed anti-*Leishmania* vaccines, appears more advantageous because it was obtained from proteins of different *Leishmania* species. Our approach is different from other vaccine candidates due to the use of species-specific proteins, as previously reported.^77,78,110^ Thus, it is expected that GH_18_-cp*Leish* may have immunogenic capacity against different species, indicating a broad anti-*Leishmania* spectrum.

The antigens and immunoglobulins stimulated (Figure 6) by GH_18_-cp*Leish* appear to be superior to those of the two multiepitope vaccines against *L. donovani* and a chimeric multiepitope vaccine (CMEV) designed for the species *L. martiniquensis* and *L. orientalis*.^77,80,111^ The production of cytokines and interleukins induced by GH_18_-cp*Leish* also appears to be superior to that of other vaccines, including the previously mentioned multiepitope vaccine developed for *L. infantum*,^78^ the vaccine targeting secretory proteins of *L. donovani* amastigotes,^110^ and yet another design based on screening by combining proteomics and immunoinformatics tools for *L. donovani*.^112^ In addition, our anti-*Leishmania* design has a more remarkable ability to stimulate the expansion of various B and T cell populations in comparison to similar studies. Thus, we have found that our proposed candidate exhibits enhanced immunogenic potential based on its predicted antigenicity score. Moreover, the combinations of proteins/GH_18_ that we utilized have not been explored in any other immunoinformatics study.

Thus, the results of this study indicate a robust immunogenic response that the proposed vaccine construct can trigger. To advance the development of this vaccine construct for laboratory evaluation, we achieved an optimal level of protein/chimera expression in hosts like *K. pastoris* is crucial, even though the CAI and GC content of the gene have already been optimized. Additional *in vitro* and *in vivo* studies are still necessary to validate the use of GH_18_-cp*Leish* as an anti-*Leishmania* vaccine in the fight against leishmaniasis.

## Conclusions

In summary, the utilization of genomic data from *Leishmania* spp. and the construction of a chimeric protein, combined with immunoinformatics tools, enabled us to identify a group of candidate peptides for anti-*Leishmania* vaccines, some of which have a high potential to encode protective immunogens. Our findings indicated the antigenic efficacy and protection of a subset of the candidate peptides. However, further experimental studies are needed to validate immune responses triggered by the predicted set of peptides and ultimately foster the anti-*Leishmania* therapeutic arsenal.

## Data Availability

The data can be made available on request to the corresponding authors.

## Supporting Information Available

Multiple amino acid sequence alignment of chitinases from family 18 (GH_18_) of *Leishmania* spp. and deduced amino acid sequence of the GH_18_-cp*Leish*, prediction of the RNA secondary structure for a chimeric gene (GH_18_-cp*Leish*), prediction of the phosphorylation sites of GH_18_-cp*Leish*, B-cell epitopes from full-length proteins using ABCpred, discontinuous B-Cell epitopes from full length (with no signal peptide) GH_18_-cp*Leish*, predicted TLRs structures and Pep_1_-cp*Leish* binding mode, and root-mean-square deviations (RMSD) of the backbone of Pep_1_-cp*Leish* and TLR_1_, as well as the radius of gyration (*R*_g_), root-mean-square fluctuation (RMSF), and hydrogen bonding (H-bond) analysis of TLR_1_ from a 100 ns molecular dynamics (MD) trajectory (dock) (PDF)
Video showing molecular dynamics of the TLR1_peptide (MP4)
