# Supplementary material for: Molecular and Immunological Properties of a Chimeric Glycosyl Hydrolase 18 Based on Immunoinformatics Approaches: A Design of a New Anti-Leishmania Vaccine
Source: ACS Pharmacol Transl Sci. 2024 Dec 31;8(1):78–96. doi: 10.1021/acsptsci.4c00341 (PMC11729430; doi:10.1021/acsptsci.4c00341)
Supplement: Supplementary file 1 — pt4c00341_si_001.pdf [file pt4c00341_si_001.pdf]

SUPPORTING INFORMATION

Molecular and Immunological Properties of a Chimeric Glycosyl Hydrolase 18 Based on Immunoinformatics Approaches: A Design of a New Anti-*Leishmania* Vaccine

José Ednésio da Cruz Freire[a,], André Nogueira Cardeal dos Santos[a], Andrelina Noronha Coelho de Souza[a], Ariclécio Cunha de Oliveira[a], Roberto Nicolete[b], Bruno Lopes de Sousa[a], João Hermínio Martins da Silva[b], Yuri de Abreu Gomes Vasconcelos[a], Isaac Neto Goes da Silva[c], Paula Matias Soares[a], Maria Izabel Florindo Guedes[d], Vânia Marilande Ceccatto[a]*

[a] *Superior Institute of Biomedical Sciences, State University of Ceará, Fortaleza, Ceará, Brazil, 60714-903*
[b] *Oswaldo Cruz Foundation (Fiocruz Ceará), Eusébio, Ceará, Brazil, 61.773-270*
[c] *Department of Veterinary Sciences, State University of Ceará, Fortaleza, Ceará, Brazil, 60714-903*
[d] *Biotechnology and Molecular Biology Laboratory, State University of Ceará, Fortaleza, Ceará, Brazil, 60714-903*

**Correspondence author. E-mail: jednesio@gmail.com*

Figure S1

1a

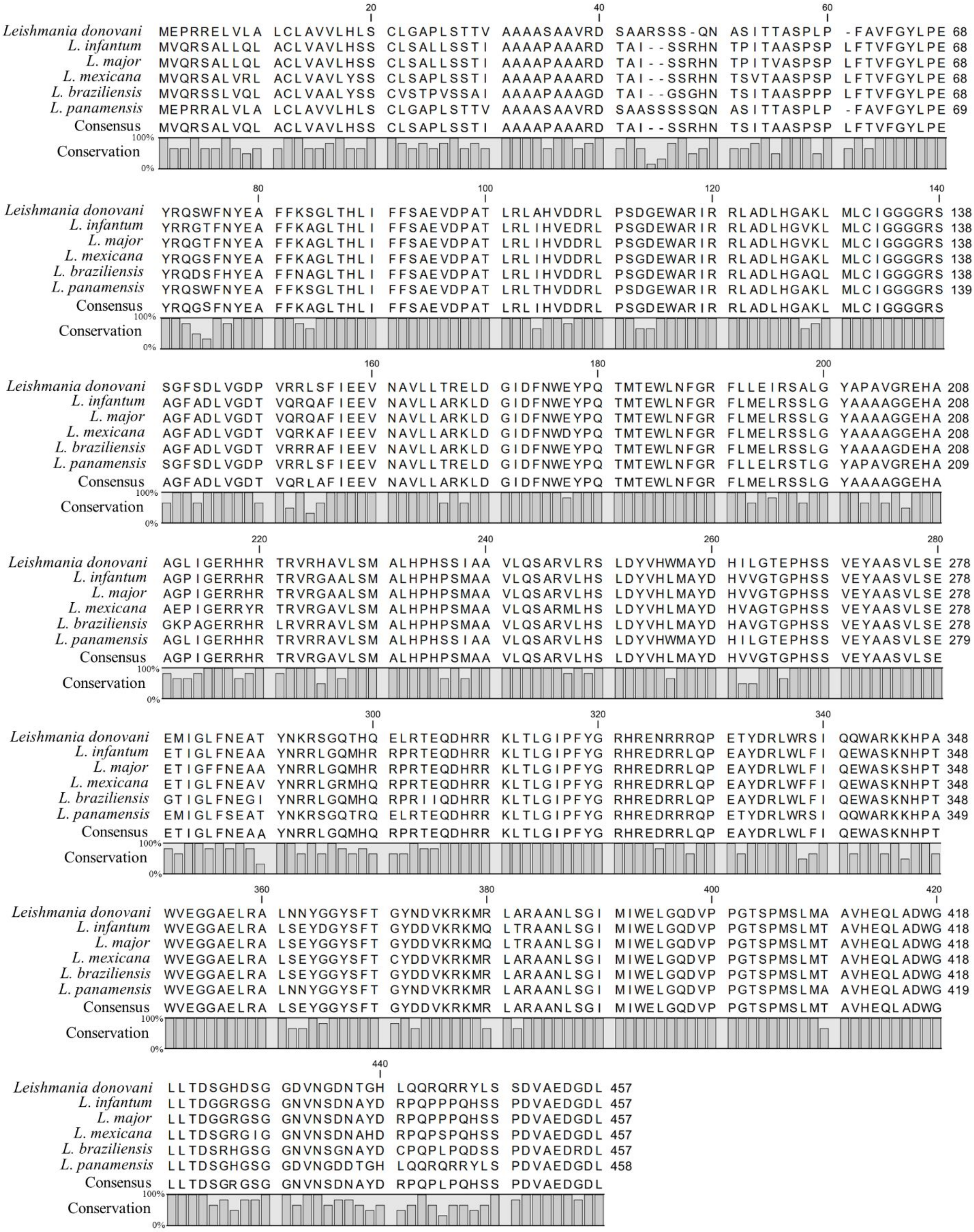
20 40 60
Leishmania donovani MEPRRELVLA LCLAVVLHLS CLGAPLSTTV AAAASAAVRD SAARSSS-QN ASITTASPLP -FAVFGYLPE 68
L. infantum MVQRSALLQL ACLVAVLHSS CLSALLSSTI AAAAPAAARD TAI--SSRHN TPITAASPSP LFTVFGYLPE 68
L. major MVQRSALLQL ACLVAVLHSS CLSALLSSTI AAAAPAAARD TAI--SSRHN TPITVASPSP LFTVFGYLPE 68
L. mexicana MVQRSALVRL ACLVAVLYSS CLSAPLSSTI AAAAPAAARD TAI--SSRHN TSVTAASPSP LFTVFGYLPE 68
L. braziliensis MVQRSSLVQL ACLVAALYSS CVSTPVSSAI AAAAPAAAGD TAI--GSGHN TSITAASPPP LFTVFGYLPE 68
L. panamensis MEPRRALVLA LCLAVVLHLS CLGAPLSTTV AAAASAAVRD SAASSSSSQN ASITTASPLP -FAVFGYLPE 69
Consensus MVQRSALVQL ACLVAVLHSS CLSAPLSSTI AAAAPAAARD TAI--SSRHN TSITAASPSP LFTVFGYLPE
100%
Conservation
0%
80 100 120 140
Leishmania donovani YRQSWFNYEA FFKSGLTHLI FFSAEVDPAT LRLAHVDDRL PSDGEWARIR RLADLHGAKL MLCIGGGGRS 138
L. infantum YRRGTFNYEA FFKAGLTHLI FFSAEVDPAT LRLIHVEDRL PSGDEWARIR RLADLHGVKL MLCIGGGGRS 138
L. major YRQGTFNYEA FFKAGLTHLI FFSAEVDPAT LRLIHVDDRL PSGDEWARIR RLADLHGVKL MLCIGGGGRS 138
L. mexicana YRQGSFNYEA FFKAGLTHLI FFSAEVDPAT LRLIHVDDRL PSGDEWARIR RLADLHGAKL MLCIGGGGRS 138
L. braziliensis YRQDSFHYEA FFNAGLTHLI FFSAEVDPAT LRLIHVDDRL PSGDEWARIR RLADLHGAQL MLCIGGGGRS 138
L. panamensis YRQSWFNYEA FFKSGLTHLI FFSAEVDPAT LRLTHVDDRL PSDGEWARIR RLADLHGAKL MLCIGGGGRS 139
Consensus YRQGSFNYEA FFKAGLTHLI FFSAEVDPAT LRLIHVDDRL PSGDEWARIR RLADLHGAKL MLCIGGGGRS
100%
Conservation
0%
160 180 200
Leishmania donovani SGFSDLVGDP VRRLSFIEEV NAVLLTRELD GIDFNWEYPQ TMTEWLNFGR FLLEIRSALG YAPAVGREHA 208
L. infantum AGFADLVGDT VQRQAFIEEV NAVLLARKLD GIDFNWEYPQ TMTEWLNFGR FLMELRSSLG YAAAAGGEHA 208
L. major AGFADLVGDT VQRQAFIEEV NAVLLARKLD GIDFNWEYPQ TMTEWLNFGR FLMELRSSLG YAAAAGGEHA 208
L. mexicana AGFADLVGDT VQRKAFIEEV NAVLLARKLD GIDFNWDYPQ TMTEWLNFGR FLMELRSSLG YAAAAGGEHA 208
L. braziliensis AGFADLVGDT VRRRAFIEEV NAVLLARKLD GIDFNWEYPQ TMTEWLNFGR FLMELRSSLG YAAAAGDEHA 208
L. panamensis SGFSDLVGDP VRRLSFIEEV NAVLLTRELD GIDFNWEYPQ TMTEWLNFGR FLLELRSTLG YAPAVGREHA 209
Consensus AGFADLVGDT VQRLAFIEEV NAVLLARKLD GIDFNWEYPQ TMTEWLNFGR FLMELRSSLG YAAAAGGEHA
100%
Conservation
0%
220 240 260 280
Leishmania donovani AGLIGERHHR TRVRHAVLSM ALHPHSSIAA VLQSARVLRS LDYVHWMAYD HILGTEPHSS VEYAASVLSE 278
L. infantum AGPIGERRHR TRVRGAALSM ALHPHPSMAA VLQSARVLHS LDYVHLMAYD HVVGTGPHSS VEYAASVLSE 278
L. major AGPIGERRHR TRVRGAALSM ALHPHPSMAA VLQSARVLHS LDYVHLMAYD HVVGTGPHSS VEYAASVLSE 278
L. mexicana AEPIGERRYR TRVRGAVLSM ALHPHPSMAA VLQSARMLHS LDYVHLMAYD HVAGTGPHSS VEYAASVLSE 278
L. braziliensis GKPAGERRHR LRVRRAVLSM ALHPHPSMAA VLQSARVLHS LDYVHLMAYD HAVGTGPHSS VEYAASVLSE 278
L. panamensis AGLIGERHHR TRVRRAVLSM ALHPHSSIAA VLQSARVLHS LDYVHWMAYD HILGTEPHSS VEYAASVLSE 279
Consensus AGPIGERRHR TRVRGAVLSM ALHPHPSMAA VLQSARVLHS LDYVHLMAYD HVVGTGPHSS VEYAASVLSE
100%
Conservation
0%
300 320 340
Leishmania donovani EMIGLFNEAT YNKRSGQTHQ ELRTEQDHRR KLTLGIPFYG RHRENRRRQP ETYDRLWRSI QQWARKKHPA 348
L. infantum ETIGLFNEAA YNRRLGQMHR RPRTEQDHRR KLTLGIPFYG RHREDRRLQP EAYDRLWLFI QEWASKNHPT 348
L. major ETIGFFNEAA YNRRLGQMHR RPRTEQDHRR KLTLGIPFYG RHREDRRLQP EAYDRLWLFI QEWASKSHPT 348
L. mexicana ETIGLFNEAV YNRRLGRMHQ RPRTEQDHRR KLTLGIPFYG RHREDRRLQP EAYDRLWFFI QEWASKNHPT 348
L. braziliensis GTIGLFNEGI YNRRLGQMHQ RPRIIQDHRR KLTLGIPFYG RHREDRRLQP EAYDRLWLFI QEWASKNHPT 348
L. panamensis EMIGLFSEAT YNKRSGQTRQ ELRTEQDHRR KLTLGIPFYG RHREDRRRQP ETYDRLWRSI QQWARKKHPA 349
Consensus ETIGLFNEAA YNRRLGQMHQ RPRTEQDHRR KLTLGIPFYG RHREDRRLQP EAYDRLWLFI QEWASKNHPT
100%
Conservation
0%
360 380 400 420
Leishmania donovani WVEGGAELRA LNNYGGYSFT GYNDVKRKMR LARAANLSGI MIWELGQDVP PGTSPMSLMA AVHEQLADWG 418
L. infantum WVEGGAELRA LSEYDGYSFT GYDDVKRKMQ LTRAANLSGI MIWELGQDVP PGTSPMSLMT AVHEQLADWG 418
L. major WVEGGAELRA LSEYGGYSFT GYDDVKRKMQ LTRAANLSGI MIWELGQDVP PGTSPMSLMT AVHEQLADWG 418
L. mexicana WVEGGAELRA LSEYGGYSFT CYDDVKRKMR LARAANLSGI MIWELGQDVP PGTSPMSLMT AVHEQLADWG 418
L. braziliensis WVEGGAELRA LSEYGGYSFT GYDDVKRKMR LARAANLSGI MIWELGQDVP PGTSPMSLMT AVHEQLADWG 418
L. panamensis WVEGGAELRA LNNYGGYSFT GYNDVKRKMR LARAANLSGI MIWELGQDVP PGTSPMSLMA AVHEQLADWG 419
Consensus WVEGGAELRA LSEYGGYSFT GYDDVKRKMR LARAANLSGI MIWELGQDVP PGTSPMSLMT AVHEQLADWG
100%
Conservation
0%
440
Leishmania donovani LLTDSGHDSG GDVNGDNTGH LQQRQRRYLS SDVAEDGDL 457
L. infantum LLTDGGRGSG GNVNSDNAYD RPQPPPQHSS PDVAEDGDL 457
L. major LLTDGGRGSG GNVNSDNAYD RPQPPPQHSS PDVAEDGDL 457
L. mexicana LLTDSGRGIG GNVNSDNAHD RPQPSPQHSS PDVAEDGDL 457
L. braziliensis LLTDSRHGSG GNVNSGNAYD CPQPLPQDSS PDVAEDRDL 457
L. panamensis LLTDSGHGSG GDVNGDDTGH LQQRQRRYLS PDVAEDGDL 458
Consensus LLTDSGRGSG GNVNSDNAYD RPQPLPQHSS PDVAEDGDL
100%
Conservation
0%

1b

**1**	atg	gtt	caa	aga	tct	gct	ttg	gtt	caa	ttg	gct	tgt	ttg	gtt	gct	gtt	ttg	cac	tct	tct	tgt	
	**M**	**V**	**Q**	**R**	**S**	**A**	**L**	**V**	**Q**	**L**	**A**	**C**	**L**	**V**	**A**	**V**	**L**	**H**	**S**	**S**	**C**	**21**
**64**	ttg	tct	gct	cca	ttg	tct	tct	act	att	gct	gct	gct	gct	cca	gct	gct	gct	aga	gac	act	gct	
	**L**	**S**	A	P	L	**S**	**S**	**T**	I	A	A	A	A	P	A	A	A	R	D	**T**	A	**42**
**127**	att	tct	tct	aga	cac	aac	act	tct	att	act	gct	gct	tct	cca	tct	cca	ttg	ttc	act	gtt	ttc	
	I	**S**	**S**	R	H	**N**	**T**	**S**	I	**T**	A	A	**S**	P	**S**	P	L	F	**T**	V	F	**63**
**190**	ggt	tac	tac	cca	gag	tac	aga	caa	ggt	tct	ttc	aac	tac	gag	gct	ttc	ttc	aag	gct	ggt	ttg	
	G	Y	Y	P	E	Y	R	Q	G	**S**	F	N	Y	E	A	F	F	K	A	G	L	**84**
**253**	act	cac	ttg	ttg	att	ttc	tct	gct	gag	gtt	gac	cca	gct	act	ttg	aga	ttg	att	act	gtt	gac	
	**T**	H	L	L	I	F	**S**	A	E	V	D	P	A	**T**	L	R	L	I	**T**	V	D	**105**
**316**	gac	aga	ttg	cca	tct	ggt	gac	gag	tgg	gct	aga	att	aga	aga	ttg	gct	gac	ttg	cac	ggt	gtt	
	D	R	L	P	**S**	G	D	E	W	A	R	I	R	R	L	A	D	L	H	G	V	**126**
**379**	aag	ttg	atg	ttg	tgt	tgt	ggt	ggt	ggt	ggt	aga	tct	gct	ggt	ttc	gct	gac	ttg	gtt	ggt	gac	
	K	L	M	L	**C**	**C**	G	G	G	G	R	**S**	A	G	F	A	D	L	V	G	D	**147**
**442**	act	gtt	caa	aga	ttg	ttg	ttc	att	gag	gag	gtt	aac	gct	gtt	ttg	ttg	act	act	aag	ttg	gac	
	**T**	V	Q	R	L	L	F	I	E	E	V	N	A	V	L	L	**T**	**T**	K	L	**D**	**168**
**505**	ggt	att	gac	ttc	aac	tgg	gag	tac	cca	caa	act	atg	act	gag	tgg	ttg	aac	ttc	ggt	aga	ttc	
	G	I	**D**	F	**N**	W	**E**	Y	P	Q	**T**	M	**T**	E	W	L	N	F	G	R	F	**189**
**568**	ttg	atg	gag	ttg	aga	tct	tct	ttg	ggt	tac	gct	gct	gct	gct	ggt	ggt	gag	cac	gct	gct	ggt	
	L	M	E	L	R	**S**	**S**	L	G	Y	A	A	A	A	G	G	E	H	A	A	G	**210**
**631**	cca	att	ggt	gag	aga	aga	cac	aga	act	aga	gtt	aga	ggt	gct	gtt	ttg	tct	atg	gct	ttg	cac	
	P	I	G	E	R	R	H	R	**T**	R	V	R	G	A	V	L	**S**	M	A	L	H	**231**
**693**	cca	cac	cac	tct	atg	gct	gct	gtt	ttg	caa	tct	gct	aga	gtt	ttg	cac	tct	ttg	gac	tac	gtt	
	P	H	H	**S**	M	A	A	V	L	Q	**S**	A	R	V	L	H	**S**	L	D	Y	V	**252**
**757**	cac	ttg	atg	gct	tac	gac	cac	gtt	gtt	ggt	act	ggt	cca	cac	tct	tct	gtt	gag	tac	gct	gct	
	H	L	M	A	Y	D	H	V	V	G	**T**	G	P	H	**S**	**S**	V	E	Y	A	A	**273**
**820**	tct	gtt	ttg	tct	gag	gag	act	att	ggt	ttg	ttc	aac	gag	gct	gct	tac	aac	aga	aga	ttg	ggt	

	**S**	V	L	**S**	E	E	**T**	I	G	L	F	N	E	A	A	Y	N	R	R	L	G	**294**
**883**	caa	atg	cac	caa	aga	cca	aga	act	gag	caa	gac	cac	aga	aga	aag	ttg	act	ttg	ggt	att	cca	
	Q	M	H	Q	R	P	R	**T**	E	Q	D	H	R	R	K	L	**T**	L	G	I	P	**315**
**946**	ttc	tac	ggt	aga	cac	aga	gag	gac	aga	aga	ttg	caa	cca	gag	gct	tac	gac	aga	ttg	tgg	ttg	
	F	Y	G	R	H	R	E	D	R	R	L	Q	P	E	A	Y	D	R	L	W	L	**336**
**1009**	ttc	att	caa	gag	tgg	gct	tct	aag	aac	cac	cca	act	tgg	gtt	gag	ggt	ggt	gct	gag	ttg	aga	
	F	I	Q	E	W	A	**S**	K	N	H	P	**T**	W	V	E	G	G	A	E	L	R	**357**
**1072**	gct	ttg	tct	gag	tac	ggt	ggt	tac	tct	ttc	act	tgt	tac	gac	gac	gtt	aag	aga	aag	atg	aga	
	A	L	**S**	E	Y	G	G	Y	**S**	F	**T**	**C**	Y	D	D	V	K	R	K	M	R	**378**
**1135**	ttg	gct	aga	gct	gct	aac	ttg	tct	ggt	att	atg	att	tgg	gag	ttg	ggt	caa	gac	gtt	cca	cca	
	L	A	R	A	A	**N**	L	**S**	G	I	M	I	W	E	L	G	Q	D	V	P	P	**399**
**1198**	ggt	act	tct	cca	atg	tct	ttg	atg	act	gct	gtt	cac	gag	caa	ttg	gct	gac	tgg	ggt	ttg	ttg	
	G	**T**	**S**	P	M	**S**	L	M	**T**	A	V	H	E	Q	L	A	D	W	G	L	L	**420**
**1261**	act	gac	tct	ggt	aga	ggt	tct	ggt	ggt	aac	gtt	aac	tct	gac	aac	gct	tac	gac	tgt	cca	caa	
	**T**	D	**S**	G	R	G	**S**	G	G	N	V	N	**S**	D	N	A	Y	D	**C**	P	Q	**441**
**1324**	cca	ttg	cca	caa	cac	tct	tct	tct	gac	gtt	gct	gag	gac	ggt	gac	ttg						
	P	L	P	Q	H	**S**	**S**	**S**	D	V	A	E	D	G	D	L						**457**

Fig. S1. Multiple amino acid sequence alignment of chitinases from family 18 (GH$_{18}$) of *Leishmania* spp. **A** – Multiple amino acid sequence alignment highlighting the consensus sequence (GH$_{18}$-cp*Leish*) using CLC Sequence Viewer v. 7.7.1. B – The deduced amino acid sequence of the GH$_{18}$-cp*Leish* is shown below the cDNA sequence. Numbers for the first nucleotide and the last amino acid residue in each row are shown on the left and right, respectively. The *N*-terminal signal peptide, as predicted by the SignalP 6.0 Server (https://services.healthtech.dtu.dk/services/SignalP-6.0/), is shown in bold. Positions containing the residues highlighted in blue and red are likely to be exposed to the *N*-glycosylation and *O*-glycosylation machinery, respectively. Positions containing the residues highlighted in orange are likely to be exposed to the formed cysteines (CyS-SCy). Positions containing the residues highlighted in green form the catalytic site.

Figure S2

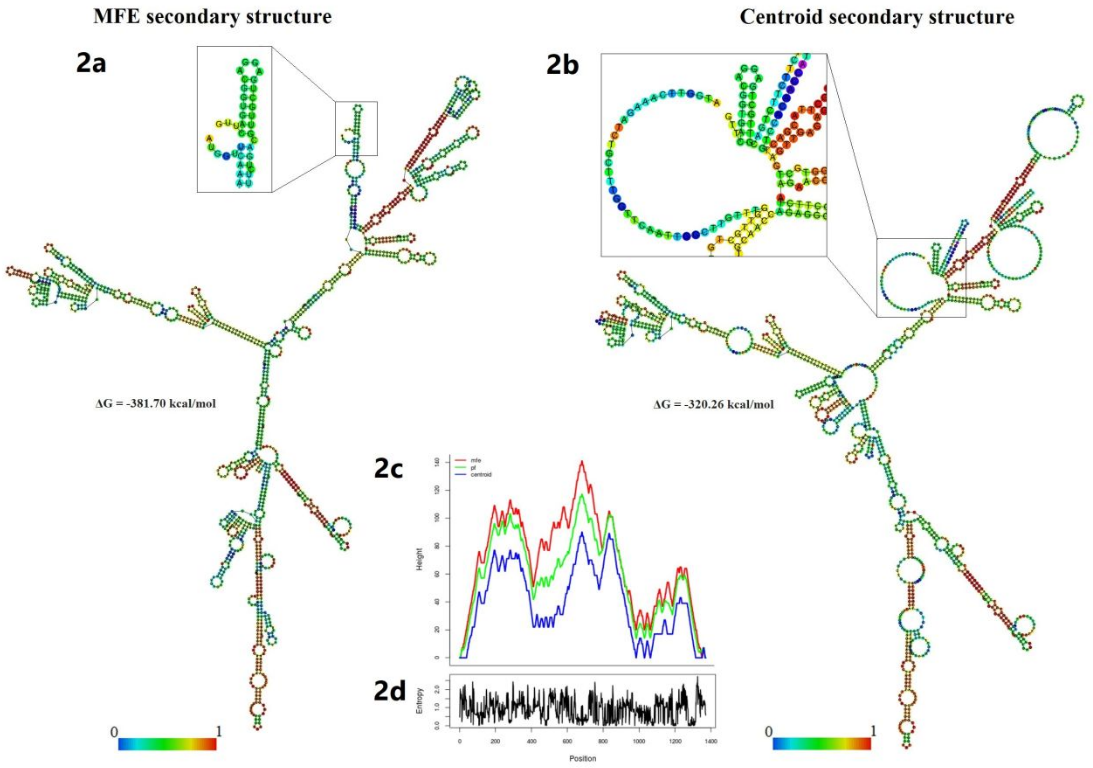

Fig. S2. Prediction of the RNA secondary structure for a chimeric gene (GH_{18}-cp*Leish*) was performed using the Vienna RNA Secondary Structure Prediction version 2.4.18 Server (http://rna.tbi.univie.ac.at/). The results include: (**a**) Secondary structure with a minimum free energy (MFE) ΔG. (**b**) Centroid secondary structure with the minimum free energy. (**c**) Mountain plot representation of the MFE structure. (**d**) Positional entropy for each position. In the mountain plot, the color gradation from blue to red represents the base-pairing probability, ranging from 0 to 1.

Table S1

Tab. S1. Prediction of the phosphorylation sites of GH$_{18}$-cp*Leish*. Residues having a prediction score above the threshold are indicated by 'S' or 'T', respectively (http://phostryp.bio.uniroma2.it/). Phosphosites are marked in red. Sites with scores below the cutoff are in blue. By default, all the sites are sorted by score. PhosTryp is a web tool trained to find phosphoproteomic sites in *Leishmania* spp, *Trypanosoma brucei*, and *T. cruzi*.

Peptide	Residue	Score
---SI**T**AASPS	3	0.834
LPQHS**S**SDVAE	399	0.805
DSGRG**S**GGNVN	378	0.759
GGGGR**S**AGFAD	89	0.751
PLPQH**S**SSDVA	398	0.735
PQHSS**S**DVAED	400	0.727
DDRLP**S**GDEWA	61	0.725
SITAA**S**PSPLF	6	0.694
TAASP**S**PLFTV	8	0.686
GGNVN**S**DNAYD	384	0.615
-----**S**ITAAS	1	0.607
GLLTD**S**GRGSG	374	0.580
VPPGT**S**PMSLM	353	0.550
DHVVG**T**GPHSS	214	0.536
EVDPA**T**LRLIT	49	0.528
VEYAA**S**VLSEE	225	0.509
MELRS**S**LGYAA	147	0.490
GTGPH**S**SVEYA	218	0.485
AASVL**S**EETIG	228	0.484
EYRQG**S**FNYEA	24	0.461
VLSEE**T**IGLFN	231	0.461
HQRPR**T**EQDHR	253	0.435
DWGLL**T**DSGRG	372	0.428
SKNHP**T**WVEGG	299	0.413
DVPPG**T**SPMSL	352	0.406

Peptide	Residue	Score
DLVGD**T**VQRLL	99	0.404
ERRHR**T**RVRGA	170	0.361
PMSLM**T**AVHEQ	359	0.353
TGPHS**S**VEYAA	219	0.348
AAVLQ**S**ARVLH	193	0.324
WEYPQ**T**MTEWL	130	0.324
ELRAL**S**EYGGY	311	0.312
RAANL**S**GIMIW	337	0.290
NAVLL**T**TKLDG	115	0.268
IQEWA**S**KNHPT	294	0.267
GTSPM**S**LMTAV	356	0.217
HRRKL**T**LGIPF	262	0.199
LHPHH**S**MAAVL	186	0.176
ARVLH**S**LDYVH	199	0.170
GGYSF**T**CYDDV	319	0.163
TLRLI**T**VDDRL	54	0.157
HLLIF**S**AEVDP	42	0.153
YPQTM**T**EWLNF	132	0.142
EYGGY**S**FTCYD	317	0.133
LMELR**S**SLGYA	146	0.128
AVLLT**T**KLDGI	116	0.119
RGAVL**S**MALHP	178	0.077
FKAGL**T**HLLIF	36	0.014
PSPLF**T**VFGYY	12	-0.047

Table S2

Tab. S2. B-Cell epitopes from full length proteins using ABCpred.

Rank	Peptide	Score	Mass (kDa)	pI	II	GRAVY
1	^{402}RGSGGNVNSDNAYDCP^{418}	0,95	16.25	4.21	33.31	-1.200
1	^{148}DFNWEYPQTMTEWLNF^{164}	0,95	21.21	3.57	17.09	-0.988
2	^{364}GIMIWELGQDVPPGTS^{380}	0,88	16.99	3.67	34.88	0.100
2	^{343}SFTCYDDVKRKMRLAR^{359}	0,88	19.89	9.78	58.51	-0.881
2	^{268}RRLGQMHQRPRTEQDH^{283}	0,88	20.45	11.52	124.41	-2.431
3	^{317}EWASKNHPTWVEGGAE^{333}	0,87	17.97	4.75	-23.51	-1.188
3	^{274}HQRPRTEQDHRRKLTL^{290}	0,87	20.71	11.54	83.31	-2.356
3	^{236}HVVGTGPHSSVEYAAS^{252}	0,87	15.97	5.98	20.29	-0.031
3	^{190}GERRHRTRVRGAVLSM^{206}	0,87	18.81	12.18	41.89	-0.975
4	^{395}GLLTDSGRGSGGNVNS^{411}	0,86	14.90	5.84	18.51	-0.519
4	^{389}EQLADWGLLTDSGRGS^{405}	0,86	17.04	4.03	16.36	-0.606
4	^{230}HLMAYDHVVGTGPHSS^{246}	0,86	17.07	6.25	28.01	-0.200
5	^{4}SSTIAAAAPAAARDTA^{19}	0,84	14.44	5.55	36.09	0.394
5	^{18}TAISSRHNTSITAASP^{34}	0,84	16.13	9.44	82.44	-0.231
6	^{140}LTTKLDGIDFNWEYPQ^{156}	0,83	19.40	4.03	7.32	-0.756
7	^{418}QPLPQHSSSDVAEDGD^{434}	0,8	16.81	3.84	81.60	-1.275
7	^{37}FTVFGYYPEYRQGSFN^{53}	0,8	19.75	6.00	31.82	-0.637
7	^{354}MRLARAANLSGIMIWE^{370}	0,8	18.32	9.35	-0.64	0.481
7	^{309}DRLWLFIQEWASKNHP^{324}	0,8	20.40	6.75	18.63	-0.819
7	^{154}PQTMTEWLNFGRFLME^{170}	0,8	20.00	4.53	20.58	-0.362
8	^{290}GIPFYGRHREDRRLQP^{306}	0,79	19.97	10.67	116.95	-1.619
9	^{323}HPTWVEGGAELRALSE^{339}	0,78	17.51	4.75	19.36	-0.475
9	^{298}REDRRLQPEAYDRLWL^{314}	0,78	21.16	6.22	128.38	-1.631
9	^{252}VLSEETIGLFNEAAYN^{267}	0,78	17.69	3.67	41.79	0.125
9	^{105}LMLCCGGGGRSAGFAD^{121}	0,78	15.14	5.82	54.67	0.631
10	^{68}SAEVDPATLRLITVDD^{84}	0,77	17.14	3.84	14.66	0.113
11	^{11}APAAARDTAISSRHNT^{27}	0,76	16.38	9.65	69.66	-0.644
12	^{85}LPSGDEWARIRRLADL^{101}	0,75	18.68	6.12	45.16	-0.512
12	^{58}KAGLTHLLIFSAEVDP^{74}	0,75	17.10	5.32	-0.02	0.556
12	^{371}GQDVPPGTSPMSLMTA^{387}	0,75	15.88	3.80	88.59	-0.125
12	^{209}PHHSMAAVLQSARVLH^{225}	0,75	17.54	10.18	64.92	0.156

12	^{201}AVLSMALHPHHSMAAV^{217}	0,75	16.72	7.06	23.00	0.887
13	^{172}SSLGYAAAAGGEHAAG^{187}	0,72	13.89	5.22	29.19	0.212
14	^{78}LITVDDRLPSGDEWAR^{94}	0,7	18.43	4.23	0.56	-0.518
14	^{383}LMTAVHEQLADWGLLT^{399}	0,7	17.98	4.35	7.83	0.531
15	^{48}QGSFNYEAFFKAGLTH^{64}	0,69	18.16	6.75	-4.73	-0.338
16	^{245}SVEYAASVLSEETIGL^{261}	0,68	16.67	3.67	56.59	0.550
16	^{24}HNTSITAASPSPLFTV^{40}	0,68	16.42	6.74	85.83	0.281
16	^{122}VGDTVQRLLFIEEVNA^{138}	0,68	18.03	4.14	42.51	0.388
17	^{144}RSAGFADLVGDTVQRL^{130}	0,66	17.04	5.96	16.83	0.037
18	^{262}NEAAYNRRLGQMHQRP^{278}	0,62	18.44	10.74	85.71	-1.773
19	^{92}ARIRRLADLHGVKLML^{108}	0,56	18.62	11.17	26.28	0.306

Table S3

Tab. S3. Discontinuous B-Cell epitopes from full length (with no signal peptide) GH_{18}-cp*Leish* using ElliPro Server.

Rank	Residues (Amino acids)	Score
1	H^{423}, S^{424}, S^{425}, S^{426}, D^{427}	0,99
2	A^{319}, S^{320}, H^{323}	0,959
3	W^{318}, K^{321}, N^{322}	0,957
4	V^{428}, A^{429}, E^{430}, D^{431}, G^{432}, D^{433}	0,944
5	N^{267}, L^{270}, G^{271}, Q^{272}, M^{273}	0,936
6	S^{410}, D^{411}, N^{412}, A^{413}	0,893
7	$R^{27}6$, P^{277}, E^{280}	0,869
8	P^{419}, L^{420}, P^{421}	0,86
9	D^{415}, C^{416}, P^{417}, Q^{418}	0,851
10	A^{1}, P^{2}, L^{3}, S^{4}, S^{5}, T^{6}, I^{7}, A^{8}, A^{9}, A^{10}, A^{11}, P^{12}, A^{13}, A^{14}, A^{15}, R^{16}, D^{17}, T^{18}, A^{19}, I^{20}, S^{21}, S^{22}, R^{23}, H^{184}, A^{185}, A^{186}, G^{187}, P^{188}, I^{189}	0,811
11	D^{300}, R^{302}, L^{303}	0,798
12	T^{156}, T^{158}, E^{159}	0,75
13	G^{190}, E^{191}, R^{193}	0,719
14	G^{259}, L^{260}, N^{262}, E^{263}	0,688
15	V^{71}, D^{72}, P^{73}, A^{74}, T^{75}, L^{76}, S^{115}, A^{116}, G^{117}, L^{121}, V^{122}, G^{123}, D^{124}, T^{125}, V^{126}, Q^{127}, R^{128}, N^{162}, R^{165}	0,675
16	M^{157}, A^{215}, V^{216}, Q^{218}, R^{221}, A^{264}, A^{265}, Y^{266}, R^{268}	0,668
17	V^{238}, G^{239}, T^{240}, G241, P^{242}, H^{297}, R^{298}, Y^{339}, G^{340}, G^{341}, Y^{342}	0,653
18	S^{254}, E^{255}, E^{256}, T^{257}	0,643
19	W^{326}, V^{327}, G^{329}, G^{330}, A^{331}, E^{332}, R^{334}, C^{346}	0,64
20	L^{397}, T^{398}, D^{399}, S^{400}, G^{401}, G^{403}, S^{404}, G^{405}, G^{406}, N^{407}, V^{408}, N^{409}	0,639
21	P^{375}, P^{376}, G^{377}, T^{378}, S^{379}, P^{380}	0,604
22	T^{26}, S^{27}, I^{28}	0,571
23	P^{86}, S^{87}, G^{88}, D^{89}, E^{90}	0,55
24	A^{180}, G^{181}, G^{182}, E^{183}	0,549
25	E^{70}, I^{79}, T^{80}	0,515
26	P^{305}, E^{306}, A^{307}, R^{310}	0,502

Table S4

Tab. S4-1. Predicted TLR_1 (PDB ID: 6NIH) structure and Pep_1-cp*Leish* binding mode.

**Hydrophobic Interatiosns (5 A)**			
**Resídue**	**Chain**	**Resídue**	**Chain**
LEU^{157}	TLR_1 (Chain B)	VAL^{408}	Pep_1-cp*Leish*
LEU^{157}	TLR_1 (Chain B)	TYR^{414}	Pep_1-cp*Leish*
LEU^{157}	TLR_1 (Chain B)	PRO^{417}	Pep_1-cp*Leish*

**Protein-Protein Ionic Interactions (7 A)**			
**Resídue**	**Chain**	**Resídue**	**Chain**
LYS^{133}	TLR_1 (Chain B)	ASP^{411}	Pep_1-cp*Leish*
ASP^{186}	TLR1 (Chain B)	ARG^{402}	Pep_1-cp*Leish*

**Protein-Protein Main Chain::Main Chain Hydrogen Bonds**										
**Donor**			**Acceptor**			**Parameters**				
**Chain**	**Resídue**	**Atom**	**Chain**	**Resídue**	**Atom**	**MO**	**Dd-a**	**Dh-a**	**A(d-H-N)**	**A(a-O=C)**
Pep_1-cp*Leish*	ARG^{402}	N	TLR_1 (Chain A)	LYS^{245}	O	-	3.34	9.99	999.99	999.99
Pep_1-cp*Leish*	GLY^{403}	N	TLR_1 (Chain A)	PRO^{244}	O	-	2.81	2.40	104.30	174.29
Pep_1-cp*Leish*	SER^{404}	N	TLR1 (Chain A)	PRO^{244}	O	-	3.08	2.11	161.72	127.62

Protein-Protein Main Chain::Side Chain Hydrogen Bonds

Donor			Acceptor			Parameters				
**Chain**	**Resídue**	**Atom**	**Chain**	**Resídue**	**Atom**	**MO**	**Dd-a**	**Dh-a**	**A(d-H-N)**	**A(a-O=C)**
TLR_1 (Chain A)	TRP^{274}	NE1	Pep_1-cp*Leish*	GLY^{403}	O	-	2.93	2.28	129.07	110.82
TLR_1 (Chain B)	LYS^{133}	NZ	Pep_1-cp*Leish*	SER^{410}	O	-	2.66	9.99	999.99	136.46
TLR_1 (Chain B)	LYS^{153}	NZ	Pep_1-cp*Leish*	CYS^{416}	O	-	2.63	9.99	999.99	111.32
TLR_1 (Chain B)	LYS^{153}	NZ	Pep_1-cp*Leish*	PRO^{417}	O	-	2.54	9.99	999.99	134.15
TLR_1 (Chain B)	ASP^{186}	OD1	Pep_1-cp*Leish*	PRO^{417}	O	1	3.04	2.01	160.69	126.82
TLR_1 (Chain B)	ASP^{186}	OD1	Pep_1-cp*Leish*	PRO^{417}	O	2	3.04	3.39	62.39	126.82
Pep_1-cp*Leish*	ARG^{402}	N	TLR_1 (Chain A)	SER^{247}	OG	-	2.97	9.99	999.99	999.99
Pep_1-cp*Leish*	ARG^{402}	NH2	TLR_1 (Chain B)	ALA^{160}	O	1	2.76	1.83	147.61	120.53
Pep_1-cp*Leish*	ARG^{402}	NH2	TLR_1 (Chain B)	ALA^{160}	O	2	2.76	3.21	55.56	120.53
Pep_1-cp*Leish*	ARG^{402}	NH2	TLR_1 (Chain B)	ASP^{186}	O	1	2.68	3.30	46.32	138.99
Pep_1-cp*Leish*	ARG^{402}	NH2	TLR_1 (Chain B)	ASP^{186}	O	2	2.68	1.78	144.21	138.99
Pep_1-cp*Leish*	GLY^{403}	N	TLR_1 (Chain A)	SER^{247}	OG	-	3.15	2.52	121.65	999.99
Pep_1-cp*Leish*	SER^{404}	OG	TLR_1 (Chain A)	PRO^{244}	O	-	3.18	9.99	999.99	102.44

Protein-Protein Side Chain::Side Chain Hydrogen Bonds

Donor			Acceptor			Parameters				
**Chain**	**Resídue**	**Atom**	**Chain**	**Resídue**	**Atom**	**MO**	**Dd-a**	**Dh-a**	**A(d-H-N)**	**A(a-O=C)**
TLR_1 (Chain B)	LYS^{133}	NZ	Pep_1-cp*Leish*	ASP^{411}	OD1	-	2.58	9.99	999.99	999.99
TLR_1 (Chain B)	LYS^{133}	NZ	Pep_1-cp*Leish*	ASP^{411}	OD2	-	2.79	9.99	999.99	999.99
TLR_1 (Chain B)	ASN^{137}	ND2	Pep_1-cp*Leish*	ASN^{409}	OD1	1	3.05	3.46	58.55	999.99

TLR_1 (Chain B)	ASN[137]	ND2	Pep_1-cp*Leish*	ASN[409]	OD1	2	3.05	2.10	154.05	999.99
TLR_1 (Chain B)	HIS[161]	NE2	Pep_1-cp*Leish*	ASN[409]	ND2	-	3.07	2.83	96.72	999.99
Pep_1-cp*Leish*	ARG[402]	NE	TLR_1 (Chain B)	ASN[188]	OD1	-	2.85	2.21	120.22	999.99
Pep_1-cp*Leish*	ASN1[409]	OD1	TLR_1 (Chain B)	ASN[137]	ND2	1	3.05	2.66	100.36	999.99
Pep_1-cp*Leish*	ASN1[409]	OD1	TLR_1 (Chain B)	ASN[137]	ND2	2	3.05	2.70	99.41	999.99
Pep_1-cp*Leish*	ASN1[409]	ND2	TLR_1 (Chain B)	HIS[161]	NE2	1	3.07	2.15	144.11	999.99
Pep_1-cp*Leish*	ASN1[409]	ND2	TLR_1 (Chain B)	HIS[161]	NE2	2	3.07	3.41	62.38	999.99

Dd-a = Distance Between Donor and Acceptor
Dh-a = Distance Between Hydrogen and Acceptor
A(d-H-N) = Angle Between Donor-H-N
A(a-O=C) = Angle Between Acceptor-O=C
MO = Multiple Occupancy
Note that angles that are undefined are written as 999.99

Tab. S4-2. Predicted TLR_2 (PDB ID: 6NIG) structure and Pep_1-cp*Leish* binding mode.

**Hydrophobic Interatiosns (5 A)**			
**TLR_2**		**Pep_1-cp*Leish***	
**Resídue**	**Chain**	**Resídue**	**Chain**
LEU^{317}	TLR_2 (Chain B)	PRO^{417}	Pep_1-cp*Leish*
ILE^{319}	TLR_2 (Chain B)	PRO^{417}	Pep_1-cp*Leish*
TYR^{323}	TLR_2 (Chain B)	PRO^{417}	Pep_1-cp*Leish*
PHE^{325}	TLR_2 (Chain A)	ALA^{413}	Pep_1-cp*Leish*
PHE^{325}	TLR_2 (Chain A)	TYR^{414}	Pep_1-cp*Leish*
PHE^{325}	TLR_2 (Chain B)	TYR^{414}	Pep_1-cp*Leish*
TYR^{326}	TLR_2 (Chain B)	PRO^{417}	Pep_1-cp*Leish*
PHE^{349}	TLR_2 (Chain A)	VAL^{408}	Pep_1-cp*Leish*
PHE^{349}	TLR_2 (Chain B)	VAL^{408}	Pep_1-cp*Leish*
LEU^{350}	TLR_2 (Chain A)	VAL^{408}	Pep_1-cp*Leish*
LEU^{350}	TLR_2 (Chain B)	VAL^{408}	Pep_1-cp*Leish*
PRO^{352}	TLR_2 (Chain B)	ALA^{413}	Pep_1-cp*Leish*

**Aromatic::Aromatic Interatiosns (7 A)**					
**Resídue**	**Chain**	**Resídue**	**Chain**	**D(centroid-centroid)**	**Dihedral Angle**
PHE^{325}	A	TYR^{414}	Pep_1-cp*Leish*	5.41	158.34
PHE^{325}	TLR_2 (Chain B)	TYR^{414}	Pep_1-cp*Leish*	4.74	24.87

Aromatic::Sulphur Interatiosns (5.3 A)					
**Resídue**	**Chain**	**Resídue**	**Chain**	**D(centroid-sulphur)**	**Angle**
PHE^{349}	TLR_2 (Chain B)	CYS^{416}	Pep_1-cp*Leish*	4.46	65.07

Cation-Pi Interatiosns (6 A)					
**Resídue**	**Chain**	**Resídue**	**Chain**	**D(cation-Pi)**	**Angle**
TYR^{323}	TLR_2 (Chain B)	ARG^{402}	Pep_1-cp*Leish*	4.42	63.06
TYR^{376}	TLR_2 (Chain A)	ARG^{402}	Pep_1-cp*Leish*	5.51	53.59

Protein-Protein Main Chain::Main Chain Hydrogen Bonds										
**Main Chain::Main chain Type**										
**Donor**			**Acceptor**				**Parameters**			
**Chain**	**Resídue**	**Atom**	**Chain**	**Resídue**	**Atom**	**MO**	**Dd-a**	**Dh-a**	**A(d-H-N)**	**A(a-O=C)**
Pep_1-cp*Leish*	ARG^{402}	N	TLR_2 (Chain B)	TYR^{323}	O	-	3.11	9.99	999.99	999.99

Protein-Protein Main Chain::Side Chain Hydrogen Bonds

Donor			Acceptor				Parameters			
**Chain**	**Resídue**	**Atom**	**Chain**	**Resídue**	**Atom**	**MO**	**Dd-a**	**Dh-a**	**A(d-H-N)**	**A(a-O=C)**
TLR_2 (Chain A)	PHE^{325}	N	Pep_1-cp*Leish*	ASP^{411}	OD2	-	3.38	4.07	39.73	97.99

TLR_2 (Chain A)	TYR^{326}	OH	Pep_1-cp*Leish*	SER^{410}	O	-	2.75	9.99	999.99	109.83
TLR_2 (Chain A)	TYR^{332}	OH	Pep_1-cp*Leish*	SER^{404}	O	-	2.95	9.99	999.99	95.61
TLR_2 (Chain A)	PHE^{349}	N	Pep_1-cp*Leish*	ASN^{409}	OD1	-	2.79	1.86	158.40	124.59
TLR_2 (Chain A)	LEU^{350}	N	Pep_1-cp*Leish*	ASN^{409}	OD1	-	2.87	2.07	137.26	132.50
TLR_2 (Chain B)	LEU^{328}	N	Pep_1-cp*Leish*	ASP^{415}	OD1	-	3.07	2.22	142.97	113.35
TLR_2 (Chain B)	PHE^{349}	N	Pep_1-cp*Leish*	CYS^{416}	SG	-	3.97	3.09	149.83	999.99
TLR_2 (Chain B)	LEU^{350}	N	Pep_1-cp*Leish*	CYS^{416}	SG	-	3.94	3.40	116.39	999.99
Pep_1-cp*Leish*	ARG^{402}	N	TLR_2 (Chain A)	ASN^{379}	OD1	-	3.37	9.99	999.99	93.25
Pep_1-cp*Leish*	ARG^{402}	N	TLR_2 (Chain A)	ASN^{379}	ND2	-	3.24	9.99	999.99	96.97
Pep_1-cp*Leish*	GLY^{406}	N	TLR_2 (Chain A)	TYR^{332}	OH	-	3.01	2.42	119.54	999.99
Pep_1-cp*Leish*	ASN^{407}	N	TLR_2 (Chain A)	TYR^{332}	OH	-	3.35	2.87	112.45	999.99
Pep_1-cp*Leish*	ASN^{409}	ND2	TLR_2 (Chain A)	LEU^{350}	O	1	2.87	1.82	173.79	148.42
Pep_1-cp*Leish*	ASN^{409}	ND2	TLR_2 (Chain A)	LEU^{350}	O	2	2.87	3.51	44.61	148.42
Pep_1-cp*Leish*	ASP^{411}	OD2	TLR_2 (Chain A)	LEU^{324}	O	1	3.16	2.98	89.82	85.42
Pep_1-cp*Leish*	ASP^{411}	OD2	TLR_2 (Chain A)	LEU^{324}	O	2	3.16	2.94	91.34	85.42
Pep_1-cp*Leish*	ASN^{412}	N	TLR_2 (Chain A)	TYR^{323}	OH	-	3.44	3.02	107.78	999.99

Protein-Protein Side Chain::Side Chain Hydrogen Bonds

Donor			Acceptor			Parameters				
**Chain**	**Resídue**	**Atom**	**Chain**	**Resídue**	**Atom**	**MO**	**Dd-a**	**Dh-a**	**A(d-H-N)**	**A(a-O=C)**
TLR_2 (Chain A)	TYR^{323}	OH	Pep_1-cp*Leish*	ASN^{412}	OD1	-	2.72	9.99	999.99	999.99
TLR_2 (Chain A)	ASP^{327}	OD1	Pep_1-cp*Leish*	ASN^{407}	ND2	1	2.85	2.81	80.86	999.99

TLR_2 (Chain A)	ASP^{327}	OD1	Pep_1-cp*Leish*	ASN^{407}	ND2	2	2.85	3.45	48.31	999.99
TLR_2 (Chain A)	ASP^{327}	OD2	Pep_1-cp*Leish*	ASN^{407}	ND2	1	2.91	2.89	80.46	999.99
TLR_2 (Chain A)	ASP^{327}	OD2	Pep_1-cp*Leish*	ASN^{407}	ND2	2	2.91	3.54	47.43	999.99
TLR_2 (Chain A)	TYR^{332}	OH	Pep_1-cp*Leish*	ASN^{407}	OD1	-	2.83	9.99	999.99	999.99
TLR_2 (Chain B)	TYR^{332}	OH	Pep_1-cp*Leish*	ASP^{415}	OD2	-	2.88	9.99	999.99	999.99
TLR_2 (Chain B)	ASN^{379}	OD1	Pep_1-cp*Leish*	ASN^{412}	ND2	1	2.85	3.47	47.02	999.99
TLR_2 (Chain B)	ASN^{379}	OD1	Pep_1-cp*Leish*	ASN^{412}	ND2	2	2.85	2.19	119.07	999.99
Pep_1-cp*Leish*	ARG^{402}	NE	TLR_2 (Chain B)	TYR^{323}	OH	-	2.96	2.33	120.05	999.99
Pep_1-cp*Leish*	ARG^{402}	NH2	TLR_2 (Chain B)	TYR^{323}	OH	1	2.68	1.84	135.32	999.99
Pep_1-cp*Leish*	ARG^{402}	NH2	TLR_2 (Chain B)	TYR^{323}	OH	2	2.68	3.46	34.90	999.99
Pep_1-cp*Leish*	ASN^{407}	ND2	TLR_2 (Chain A)	ASP^{327}	OD1	1	2.85	2.18	119.09	999.99
Pep_1-cp*Leish*	ASN^{407}	ND2	TLR_2 (Chain A)	ASP^{327}	OD1	2	2.85	2.95	74.13	999.99
Pep_1-cp*Leish*	ASN^{407}	ND2	TLR_2 (Chain A)	ASP^{327}	OD2	1	2.91	2.78	86.41	999.99
Pep_1-cp*Leish*	ASN^{407}	ND2	TLR_2 (Chain A)	ASP^{327}	OD2	2	2.91	2.31	116.15	999.99
Pep_1-cp*Leish*	ASN^{407}	OD1	TLR_2 (Chain A)	TYR^{332}	OH	1	2.83	2.67	87.32	999.99
Pep_1-cp*Leish*	ASN^{407}	OD1	TLR_2 (Chain A)	TYR^{332}	OH	2	2.83	2.63	90.12	999.99
Pep_1-cp*Leish*	ASN^{412}	OD1	TLR_2 (Chain A)	TYR^{323}	OH	1	2.72	3.14	57.56	999.99
Pep_1-cp*Leish*	ASN^{412}	OD1	TLR_2 (Chain A)	TYR^{323}	OH	2	2.72	2.82	73.70	999.99
Pep_1-cp*Leish*	ASN^{412}	ND2	TLR_2 (Chain B)	ASN^{379}	OD1	1	2.85	3.47	46.27	999.99
Pep_1-cp*Leish*	ASN^{412}	ND2	TLR_2 (Chain B)	ASN^{379}	OD1	2	2.85	2.03	135.39	999.99
Pep_1-cp*Leish*	TYR^{414}	OH	TLR_2 (Chain A)	ASP^{327}	OD2	-	2.80	9.99	999.99	999.99

Dd-a = Distance Between Donor and Acceptor

Dh-a = Distance Between Hydrogen and Acceptor

A(d-H-N) = Angle Between Donor-H-N

A(a-O=C) = Angle Between Acceptor-O=C

MO = Multiple Occupancy

Note that angles that are undefined are written as 999.99

Tab. S4-3. Predicted TLR_3 (PDB ID: 7WFI) structure and Pep_1-cp*Leish* binding mode.

Hydrophobic Interatiosns (5 A)			
TLR_3		Pep_1-cp*Leish*	
Resídue	Chain	Resídue	Chain
LEU^{595}	TLR_3 (Chain B)	VAL^{408}	Pep_1-cp*Leish*
LEU^{621}	TLR_3 (Chain B)	VAL^{408}	Pep_1-cp*Leish*
LEU^{676}	TLR_3 (Chain B)	PRO^{417}	Pep_1-cp*Leish*

Protein-Protein Ionic Interactions (7 A)			
Resídue	Chain	Resídue	Chain
LYS^{619}	TLR_3 (Chain B)	ASP^{415}	Pep_1-cp*Leish*
HIS^{674}	TLR_3 (Chain B)	ASP^{415}	Pep_1-cp*Leish*

Protein-Protein Main Chain::Main Chain Hydrogen Bonds										
Main Chain::Main chain Type										
Donor			Acceptor			Parameters				
Chain	Resídue	Atom	Chain	Resídue	Atom	MO	Dd-a	Dh-a	A(d-H-N)	A(a-O=C)
TLR_3 (Chain B)	Asn^{645}	N	Pep_1-cp*Leish*	Cys^{416}	O	-	3.04	3.20	72.11	168.83
Pep_1-cp*Leish*	Arg^{402}	N	TLR_3 (Chain A)	Leu^{621}	O	-	3.31	9.99	999.99	999.99
Pep_1-cp*Leish*	Gly^{403}	N	TLR_3 (Chain A)	Asn^{620}	O	-	3.27	2.53	133.42	126.98

Protein-Protein Main Chain::Side Chain Hydrogen Bonds

Donor			Acceptor				Parameters			
Chain	Resídue	Atom	Chain	Resídue	Atom	MO	Dd-a	Dh-a	A(d-H-N)	A(a-O=C)
TLR_3 (Chain A)	Leu^{621}	N	Pep_1-cp*Leish*	Ser^{404}	OG	-	3.18	3.34	72.61	999.99
TLR_3 (Chain B)	Lys^{619}	NZ	Pep_1-cp*Leish*	Asn^{412}	O	-	2.64	9.99	999.99	118.37
TLR_3 (Chain B)	Lys^{619}	NZ	Pep_1-cp*Leish*	Ala^{413}	O	-	2.51	9.99	999.99	109.86
TLR_3 (Chain B)	Lys^{619}	NZ	Pep_1-cp*Leish*	Tyr^{414}	O	-	2.57	9.99	999.99	136.55
TLR_3 (Chain B)	Asn^{678}	ND2	Pep_1-cp*Leish*	Pro^{417}	O	1	2.98	3.69	41.34	155.47
TLR_3 (Chain B)	Asn^{678}	ND2	Pep_1-cp*Leish*	Pro^{417}	O	2	2.98	2.05	151.49	155.47
Pep_1-cp*Leish*	Ser^{404}	OG	TLR_3 (Chain A)	Asn^{596}	O	-	2.69	9.99	999.99	144.45
Pep_1-cp*Leish*	Asn^{409}	ND2	TLR_3 (Chain B)	Leu^{595}	O	1	2.82	2.92	73.95	146.30
Pep_1-cp*Leish*	Asn^{409}	ND2	TLR_3 (Chain B)	Leu^{595}	O	2	2.82	2.05	129.79	146.30

Protein-Protein Side Chain::Side Chain Hydrogen Bonds

Donor			Acceptor				Parameters			
Chain	Resídue	Atom	Chain	Resídue	Atom	MO	Dd-a	Dh-a	A(d-H-N)	A(a-O=C)
TLR_3 (Chain A)	ASN^{597}	OD1	Pep_1-cp*Leish*	ASN^{407}	ND2	1	3.06	2.06	152.52	999.99
TLR_3 (Chain A)	ASN^{597}	OD1	Pep_1-cp*Leish*	ASN^{407}	ND2	2	3.06	3.46	59.11	999.99
TLR_3 (Chain A)	ASN^{597}	ND2	Pep_1-cp*Leish*	TYR^{414}	OH	1	2.80	1.84	147.95	999.99
TLR_3 (Chain A)	ASN^{597}	ND2	Pep_1-cp*Leish*	TYR^{414}	OH	2	2.80	3.34	49.92	999.99
TLR_3 (Chain B)	GLU^{570}	OE1	Pep_1-cp*Leish*	ASN^{412}	ND2	1	3.09	2.26	133.31	999.99
TLR_3 (Chain B)	GLU^{570}	OE1	Pep_1-cp*Leish*	ASN^{412}	ND2	2	3.09	3.24	72.49	999.99
TLR_3 (Chain B)	ASN^{597}	OD1	Pep_1-cp*Leish*	ASN^{409}	OD1	1	3.22	2.35	136.53	999.99

TLR_3 (Chain B)	ASN^{597}	OD1	Pep_1-cp*Leish*	ASN^{409}	OD1	2	3.22	4.11	27.57	999.99
TLR_3 (Chain B)	ASN^{597}	OD1	Pep_1-cp*Leish*	ASN^{409}	ND2	1	2.87	2.00	136.01	999.99
TLR_3 (Chain B)	ASN^{597}	OD1	Pep_1-cp*Leish*	ASN^{409}	ND2	2	2.87	3.31	56.65	999.99
TLR_3 (Chain B)	ASN^{597}	ND2	Pep_1-cp*Leish*	ASN^{409}	OD1	1	2.91	1.90	157.53	999.99
TLR_3 (Chain B)	ASN^{597}	ND2	Pep_1-cp*Leish*	ASN^{409}	OD1	2	2.91	3.67	36.07	999.99
TLR_3 (Chain B)	GLN^{618}	NE2	Pep_1-cp*Leish*	ASN^{412}	OD1	1	2.89	2.01	138.72	999.99
TLR_3 (Chain B)	GLN^{618}	NE2	Pep_1-cp*Leish*	ASN^{412}	OD1	2	2.89	3.25	60.68	999.99
Pep_1-cp*Leish*	ARG^{402}	NH1	TLR_3 (Chain A)	ASN^{678}	OD1	1	2.70	3.60	25.71	999.99
Pep_1-cp*Leish*	ARG^{402}	NH1	TLR_3 (Chain A)	ASN^{678}	OD1	2	2.70	1.98	125.45	999.99
Pep_1-cp*Leish*	ARG^{402}	NH2	TLR_3 (Chain A)	ASN^{678}	OD1	1	2.63	3.50	28.38	999.99
Pep_1-cp*Leish*	ARG^{402}	NH2	TLR_3 (Chain A)	ASN^{678}	OD1	2	2.63	1.89	126.80	999.99
Pep_1-cp*Leish*	ASN^{407}	ND2	TLR_3 (Chain A)	ASN^{597}	OD1	1	3.06	2.62	104.13	999.99
Pep_1-cp*Leish*	ASN^{407}	ND2	TLR_3 (Chain A)	ASN^{597}	OD1	2	3.06	2.76	97.22	999.99
Pep_1-cp*Leish*	ASN^{409}	OD1	TLR_3 (Chain B)	ASN^{597}	OD1	1	3.22	2.40	131.39	999.99
Pep_1-cp*Leish*	ASN^{409}	OD1	TLR_3 (Chain B)	ASN^{597}	OD1	2	3.22	4.11	27.48	999.99
Pep_1-cp*Leish*	ASN^{409}	OD1	TLR_3 (Chain B)	ASN^{597}	ND2	1	2.91	1.87	161.14	999.99
Pep_1-cp*Leish*	ASN^{409}	OD1	TLR_3 (Chain B)	ASN^{597}	ND2	2	2.91	3.40	53.75	999.99
Pep_1-cp*Leish*	ASN^{409}	ND2	TLR_3 (Chain B)	ASN^{597}	OD1	1	2.87	1.91	148.91	999.99
Pep_1-cp*Leish*	ASN^{409}	ND2	TLR_3 (Chain B)	ASN^{597}	OD1	2	2.87	3.61	37.63	999.99
Pep_1-cp*Leish*	ASN^{412}	ND2	TLR_3 (Chain B)	GLU^{570}	OE1	1	3.09	2.33	128.28	999.99
Pep_1-cp*Leish*	ASN^{412}	ND2	TLR_3 (Chain B)	GLU^{570}	OE1	2	3.09	3.14	78.24	999.99
Pep_1-cp*Leish*	ASN^{412}	OD1	TLR_3 (Chain B)	GLN^{618}	NE2	1	2.89	1.86	159.76	999.99

Pep_1-cp*Leish*	ASN^{412}	OD1	TLR_3 (Chain B)	GLN^{618}	NE2	2	2.89	3.27	59.78	999.99
Pep_1-cp*Leish*	TYR^{414}	OH	TLR_3 (Chain A)	ASN^{597}	ND2	-	2.80	9.99	999.99	999.99

Dd-a = Distance Between Donor and Acceptor
Dh-a = Distance Between Hydrogen and Acceptor
A(d-H-N) = Angle Between Donor-H-N
A(a-O=C) = Angle Between Acceptor-O=C
MO = Multiple Occupancy
Note that angles that are undefined are written as 999.99

Tab. S4-4. Predicted TLR_4 (PDB ID: 3FXI) structure and Pep_1-cp*Leish* binding mode.

**Hydrophobic Interatiosns (5 A)**			
**TLR_4**		**Pep_1-cp*Leish***	
**Resídue**	**Chain**	**Resídue**	**Chain**
PHE^{408}	TLR_4 (Chain B)	ALA^{413}	Pep_1-cp*Leish*

**Protein-Protein Ionic Interactions (7 A)**			
**Resídue**	**Chain**	**Resídue**	**Chain**
ARG^{382}	TLR_4 (Chain B)	ASP^{415}	Pep_1-cp*Leish*
LYS^{435}	TLR_4 (Chain A)	ASP^{411}	Pep_1-cp*Leish*
ASP^{502}	TLR_4 (Chain B)	ARG^{402}	Pep_1-cp*Leish*
ASP^{550}	TLR_4 (Chain B)	ARG^{402}	Pep_1-cp*Leish*

**Protein-Protein Main Chain::Main Chain Hydrogen Bonds**										
**Main Chain::Main chain Type**										
**Donor**			**Acceptor**			**Parameters**				
**Chain**	**Resídue**	**Atom**	**Chain**	**Resídue**	**Atom**	**MO**	**Dd-a**	**Dh-a**	**A(d-H-N)**	**A(a-O=C)**
TLR_4 (Chain A)	GLN^{436}	N	Pep_1-cp*Leish*	SER^{410}	O	-	2.81	1.86	161.32	155.74

**Protein-Protein Main Chain::Side Chain Hydrogen Bonds**		
**Donor**	**Acceptor**	**Parameters**

Chain	Resídue	Atom	Chain	Resídue	Atom	MO	Dd-a	Dh-a	A(d-H-N)	A(a-O=C)
TLR_4 (Chain A)	ARG^{460}	NH2	Pep_1-cp*Leish*	GLY^{403}	O	1	2.68	2.05	115.91	131.35
TLR_4 (Chain A)	ARG^{460}	NH2	Pep_1-cp*Leish*	GLY^{403}	O	2	2.68	2.66	80.35	131.35
TLR_4 (Chain A)	ARG^{460}	NE	Pep_1-cp*Leish*	GLY^{406}	O	-	2.73	2.44	96.49	141.82
TLR_4 (Chain A)	ARG^{460}	NH2	Pep_1-cp*Leish*	GLY^{406}	O	1	2.98	2.65	97.76	95.96
TLR_4 (Chain A)	ARG^{460}	NH2	Pep_1-cp*Leish*	GLY^{406}	O	2	2.98	3.71	37.92	95.96
TLR_4 (Chain A)	ARG^{460}	N	Pep_1-cp*Leish*	SER^{410}	OG	-	3.01	2.33	124.34	999.99
TLR_4 (Chain B)	GLN^{430}	OE1	Pep_1-cp*Leish*	CYS^{416}	O	1	2.89	2.31	112.32	84.59
TLR_4 (Chain B)	GLN^{430}	OE1	Pep_1-cp*Leish*	CYS^{416}	O	2	2.89	3.26	60.25	84.59
TLR_4 (Chain B)	GLN^{430}	OE1	Pep_1-cp*Leish*	PRO^{417}	OXT	1	1.79	1.09	111.87	132.14
TLR_4 (Chain B)	GLN^{430}	OE1	Pep_1-cp*Leish*	PRO^{417}	OXT	2	1.79	2.72	21.55	132.14
TLR_4 (Chain B)	GLN^{430}	NE2	Pep_1-cp*Leish*	PRO^{417}	OXT	1	2.02	1.43	107.84	151.32
TLR_4 (Chain B)	GLN^{430}	NE2	Pep_1-cp*Leish*	PRO^{417}	OXT	2	2.02	2.99	14.97	151.32
TLR_4 (Chain B)	HIS^{431}	ND1	Pep_1-cp*Leish*	ASN^{412}	O	-	2.85	2.00	156.21	148.19
TLR_4 (Chain B)	HIS^{431}	ND1	Pep_1-cp*Leish*	TYR^{414}	O	-	3.26	3.26	81.87	123.61
TLR_4 (Chain B)	LYS^{477}	NZ	Pep_1-cp*Leish*	PRO^{417}	O	-	2.56	9.99	999.99	139.02
Pep_1-cp*Leish*	SER^{404}	N	TLR_4 (Chain A)	GLU^{485}	OE1	-	3.42	3.67	67.56	114.67
Pep_1-cp*Leish*	SER^{410}	OG	TLR_4 (Chain A)	ARG^{460}	O	-	2.73	9.99	999.99	102.96
Pep_1-cp*Leish*	ASN^{412}	ND2	TLR_4 (Chain B)	ASN^{409}	O	1	3.05	3.20	72.18	168.21
Pep_1-cp*Leish*	ASN^{412}	ND2	TLR_4 (Chain B)	ASN^{409}	O	2	3.05	2.51	111.86	168.21
Pep_1-cp*Leish*	ASN^{412}	ND2	TLR_4 (Chain B)	HIS^{431}	O	1	2.80	3.54	38.79	137.55
Pep_1-cp*Leish*	ASN^{412}	ND2	TLR_4 (Chain B)	HIS^{431}	O	2	2.80	1.99	133.84	137.55

Protein-Protein Side Chain::Side Chain Hydrogen Bonds

Donor			Acceptor				Parameters			
**Chain**	**Resídue**	**Atom**	**Chain**	**Resídue**	**Atom**	**MO**	**Dd-a**	**Dh-a**	**A(d-H-N)**	**A(a-O=C)**
TLR_4 (Chain A)	LYS^{435}	NZ	Pep_1-cp*Leish*	ASP^{411}	OD1	-	2.65	9.99	999.99	999.99
TLR_4 (Chain A)	LYS^{435}	NZ	Pep_1-cp*Leish*	ASP^{411}	OD2	-	2.48	9.99	999.99	999.99
TLR_4 (Chain A)	ARG^{460}	NH1	Pep_1-cp*Leish*	ASN^{407}	OD1	1	2.59	3.41	33.24	999.99
TLR_4 (Chain A)	ARG^{460}	NH1	Pep_1-cp*Leish*	ASN^{407}	OD1	2	2.59	1.98	116.38	999.99
TLR_4 (Chain A)	ARG^{460}	NH2	Pep_1-cp*Leish*	ASN^{407}	OD1	1	2.75	3.62	28.99	999.99
TLR_4 (Chain A)	ARG^{460}	NH2	Pep_1-cp*Leish*	ASN^{407}	OD1	2	2.75	2.16	115.00	999.99
TLR_4 (Chain B)	ARG^{382}	NE	Pep_1-cp*Leish*	ASP^{415}	OD2	-	2.83	2.04	134.33	999.99
TLR_4 (Chain B)	ARG^{382}	NH2	Pep_1-cp*Leish*	ASP^{415}	OD1	1	2.81	1.83	155.85	999.99
TLR_4 (Chain B)	ARG^{382}	NH2	Pep_1-cp*Leish*	ASP^{415}	OD1	2	2.81	3.31	52.96	999.99
TLR_4 (Chain B)	ARG^{382}	NH2	Pep_1-cp*Leish*	ASP^{415}	OD2	1	2.93	2.09	136.13	999.99
TLR_4 (Chain B)	ARG^{382}	NH2	Pep_1-cp*Leish*	ASP^{415}	OD2	2	2.93	3.80	28.32	999.99
TLR_4 (Chain B)	GLN^{430}	OE1	Pep_1-cp*Leish*	CYS^{416}	SG	1	3.45	3.48	79.23	999.99
TLR_4 (Chain B)	GLN^{430}	OE1	Pep_1-cp*Leish*	CYS^{416}	SG	2	3.45	2.61	136.46	999.99
TLR_4 (Chain B)	HIS^{458}	NE2	Pep_1-cp*Leish*	ASN^{409}	ND2	-	2.96	2.13	152.97	999.99
Pep_1-cp*Leish*	ARG^{402}	NE	TLR_4 (Chain B)	GLN^{505}	OE1	-	2.88	2.30	116.46	999.99
Pep_1-cp*Leish*	ARG^{402}	NH2	TLR_4 (Chain B)	GLN^{505}	OE1	1	2.64	1.80	135.19	999.99
Pep_1-cp*Leish*	ARG^{402}	NH2	TLR_4 (Chain B)	GLN^{505}	OE1	2	2.64	3.44	32.99	999.99
Pep_1-cp*Leish*	ASN^{409}	ND2	TLR_4 (Chain B)	HIS^{458}	NE2	1	2.96	3.36	59.09	999.99
Pep_1-cp*Leish*	ASN^{409}	ND2	TLR_4 (Chain B)	HIS^{458}	NE2	2	2.96	2.31	120.65	999.99

Pep_1-cp*Leish*	CYS^{416}	SG	TLR_4 (Chain B)	GLN^{430}	OE1	-	3.45	9.99	999.99	999.99

Dd-a = Distance Between Donor and Acceptor

Dh-a = Distance Between Hydrogen and Acceptor

A(d-H-N) = Angle Between Donor-H-N

A(a-O=C) = Angle Between Acceptor-O=C

MO = Multiple Occupancy

Note that angles that are undefined are written as 999.99

Figure S3

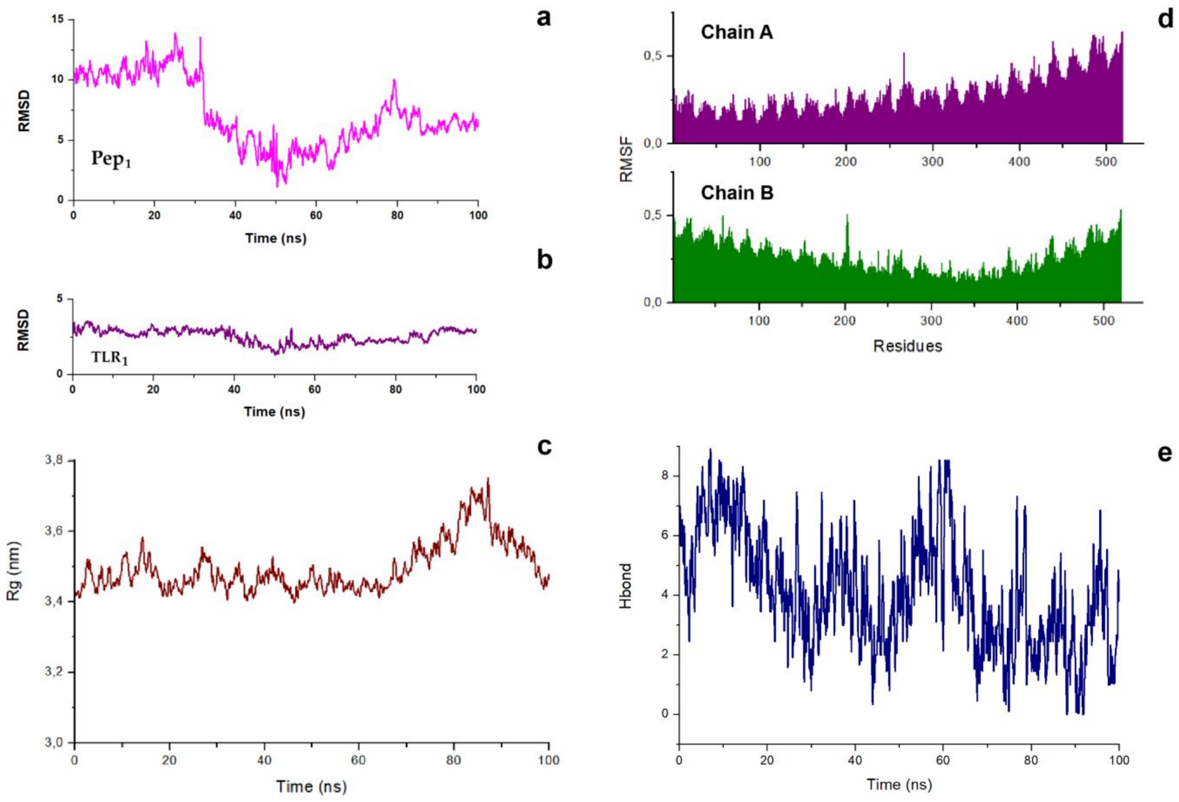

Fig. S3. Root-mean-square deviations (RMSD) of the backbone of Pep_1-cp*Leish* and TLR_1, as well as the radius of gyration (R_g), root-mean-square fluctuation (RMSF), and hydrogen bonding (H-bond) analysis of TLR_1 from a 100 ns molecular dynamics (MD) trajectory: (**a**) and (**b**) Visualizations of the RMSD of Pep_1 (pink) and TLR_1 (magenta). (**c**) Visualization of the R_g of the TLR_1 complex with Pep_1 (burgundy red). (**d**) Visualizations of the RMSF per residue for Chain A (purple) and Chain B (green) of TLR_1. (**e**) Number of hydrogen bond interactions formed in the complex between Pep_1 and TLR_1 (blue).

Table S5

Tab. S5. Short- and long-range Lennard-Jones interactions and electrostatic forces were identified in the Pep_1-cp*Leish*::TLR_1 complex. The data were generated using the Arpeggio server (https://biosig.lab.uq.edu.au/arpeggioweb/calculate/). All residues shown in bold correspond to Pep_1, while those not in bold belong to the TLR_1 receptor. The residues belonging to Pep_1-cp*Leish* (Pep_1) in a given column interact with the TLR1 residues located immediately to their left in the same row and column, respectively.

Short and long-range Lennard-Jones interactions

		**Pep_1**	**TLR_1**	**Pep_1**	**TLR_1**	**Pep_1**	**TLR_1**	**Pep_1**	**TLR_1**	**Pep_1**	**TLR_1**	**Pep_1**	**TLR_1**	**Pep_1**	**TLR_1**	**Pep_1**	**TLR_1**	**Pep_1**	**TLR_1**	**Pep_1**	**TLR_1**	**Pep_1**	**TLR_1**	**Total interactions**
**Van der Waals (VdW)**	Hbond-VdW	**Gly^{403}**	Ser^{247}	**Ser^{404}**	Pro^{244}																			**2**
	WeakHbond-VdW	**Val^{408}**	Pro^{158}	**Asn^{412}**	Leu^{157}	**Cys^{416}**	Asp^{186}																	**3**
	Polar-VdW	**Gly^{403}**	Ser^{247}	**Ser^{404}**	Pro^{244}																			**2**
	Hydrophobic-VdW	**Arg^{402}**	Trp^{274}	**Val^{408}**	Leu^{157}	**Tyr^{414}**	Leu^{157}	**Cys^{416}**	Leu^{157}															**4**
**Clashes de Van der Waals**	Hbond-vdwclash	**Arg^{402}**	Asp^{186}	**Arg^{402}**	Ala^{160}	**Arg^{402}**	Ser^{247}	**Gly^{403}**	Pro^{244}	**Ser^{404}**	Trp^{274}	**Asn^{409}**	His^{161}	**Asn^{409}**	Asn^{137}	**Ser^{410}**	Lys^{133}	**Asp^{411}**	Lys^{133}	**Cys^{416}**	Lys^{153}	**Pro^{417}**	Lys^{153}	**11**
	Polar-vdwclash	**Arg^{402}**	Ser^{247}	**Arg^{402}**	Asn^{188}	**Gly^{403}**	Pro^{244}	**Gly^{403}**	Trp^{274}	**Asn^{409}**	Asn^{137}	**Ser^{410}**	Lys^{133}	**Asp^{411}**	Lys^{133}	**Cys^{416}**	Lys^{153}	**Pro^{417}**	Lys^{153}					**9**
	Undefined-vdwclash	**Gly^{405}**	His^{161}	**Asp^{411}**	Lys^{133}																			**2**
**Proximal**	Polar-proximal	**Arg^{402}**	Lys^{245}	**Ser^{404}**																				**6**
	Hbond-proximal	**Arg^{402}**	Lys^{245}	**Ser^{404}**	Pro^{244}																			**4**
	WeakHbond-proximal	**Gly^{405}**	His^{161}	**Val^{408}**	Leu^{157}	**Asn^{412}**	Pro^{158}	**Cys^{416}**	Leu^{157}															**4**
	Hydrophobic-proximal	**Arg^{402}**	Trp^{274}	**Val^{408}**	Leu^{157}	**Tyr^{414}**	Leu^{157}	**Cys^{416}**	Leu^{157}															**8**
	Carbonyl-proximal	**Arg^{402}**	Pro^{244}																					**1**

Electrostaic force

		**Pep_1**	**TLR_1**	**Pep_1**	**TLR_1**	**Pep_1**	**TLR_1**	**Pep_1**	**TLR_1**	**Pep_1**	**TLR_1**	**Pep_1**	**TLR_1**	**Pep_1**	**TLR_1**	**Pep_1**	**TLR_1**	**Pep_1**	**TLR_1**	**Pep_1**	**TLR_1**	**Pep_1**	**TLR_1**	**Total interactions**
**H-bond**	Hbond-proximal	**Arg^{402}**	Lys^{245}	**Ser^{404}**	Pro^{244}																			**2**
	Hbond-VdW	**Gly^{403}**	Ser^{247}	**Ser^{404}**	Pro^{244}																			**2**
	Hbond-VdWclash	**Arg402**	Asp^{186}	**Arg^{402}**	Ala^{160}	**Arg^{402}**	Ser^{247}	**Gly^{403}**	Pro^{244}	**Ser^{404}**	Trp^{274}	**Asn^{409}**	His^{161}	**Asn^{409}**	Asn^{137}	**Ser^{410}**	Lys^{133}	**Asp^{411}**	Lys^{133}	**Cys^{416}**	Lys^{153}	**Pro^{417}**	Lys^{153}	**11**
**Ionic interation**	Ionic-VdWClash	**Asp^{411}**	Lys^{133}																					**1**
**Polar contact**	Polar-proximal	**Arg^{402}**	Lys^{245}	**Ser^{404}**	Pro^{244}																			**2**
	Polar-VdW	**Gly^{403}**	Ser^{247}	**Ser^{404}**	Pro^{244}																			**2**
	Weakpolar-VdW	**Gly^{403}**	Pro^{244}	**Val^{408}**	Pro^{158}	**Asn^{412}**	Leu^{157}	**Cys^{416}**	Asp^{186}															**4**
**Carbonyl**	Carbonyl-proximal	**Arg^{402}**	Pro^{244}																					**1**

Video S1

Video_TLR1_peptide.mp4

Vid. S1. Simulation between the anti-*Leishmania* Pep_1–cp*Leish* peptide and the TLR_1 receptor (Toll-like receptor 1).
